# Current Trends in Sirtuin Activator and Inhibitor Development

**DOI:** 10.3390/molecules29051185

**Published:** 2024-03-06

**Authors:** Karina L. Bursch, Christopher J. Goetz, Brian C. Smith

**Affiliations:** 1 Department of Biochemistry, Medical College of Wisconsin, Milwaukee, WI 53226, USA; kbursch@mcw.edu (K.L.B.);; 2 Structural Genomics Unit, Linda T. and John A. Mellowes Center for Genomic Sciences and Precision Medicine, Medical College of Wisconsin, Milwaukee, WI 53226, USA; 3 Program in Chemical Biology, Medical College of Wisconsin, Milwaukee, WI 53226, USA

**Keywords:** sirtuins, epigenetics, lysine deacylation, drug development

## Abstract

Sirtuins are NAD^+^-dependent protein deacylases and key metabolic regulators, coupling the cellular energy state with selective lysine deacylation to regulate many downstream cellular processes. Humans encode seven sirtuin isoforms (Sirt1-7) with diverse subcellular localization and deacylase targets. Sirtuins are considered protective anti-aging proteins since increased sirtuin activity is canonically associated with lifespan extension and decreased activity with developing aging-related diseases. However, sirtuins can also assume detrimental cellular roles where increased activity contributes to pathophysiology. Modulation of sirtuin activity by activators and inhibitors thus holds substantial potential for defining the cellular roles of sirtuins in health and disease and developing therapeutics. Instead of being comprehensive, this review discusses the well-characterized sirtuin activators and inhibitors available to date, particularly those with demonstrated selectivity, potency, and cellular activity. This review also provides recommendations regarding the best-in-class sirtuin activators and inhibitors for practical research as sirtuin modulator discovery and refinement evolve.

## 1. Introduction

The sirtuin or silent information regulator 2 (Sir2) protein family was named for the *Saccharomyces cerevisiae* Sir2 protein [1], a product of the *SIR* gene family implicated in silencing yeast gene expression [2] by facilitating a less accessible chromatin structure [3]. Since these foundational studies, Sir2 orthologs have been identified across all taxonomic domains (Archaea, Bacteria, and Eukarya) [4]. Sirtuins regulate various pro-survival, lifespan-extending cellular processes, including DNA transcription and repair, metabolism, and stress resistance [5]. Mechanistically, sirtuins are NAD^+^-dependent protein deacylases that remove acyl modifications on lysine residues of histone and non-histone protein substrates, yielding deacylated protein substrate, nicotinamide, and 2′-*O*-acyl-ADP-ribose (*O*AADPr) as products [6,7]. NAD^+^ dependence endows sirtuins with cellular ‘energy sensing’ capabilities, coupling the energetic needs of the cell with selective lysine deacylation across subcellular organelles [8]. All mammals, including humans, express seven sirtuin isoforms (Sirt1-7) [5] that comprise the class III histone deacetylase (HDAC) family [9] and display diverse subcellular localization, deacylase activities, and protein targets [10,11].

### 1.1. Sirtuin Subcellular Localization and Structure Dictate Function

**Figure 1:**
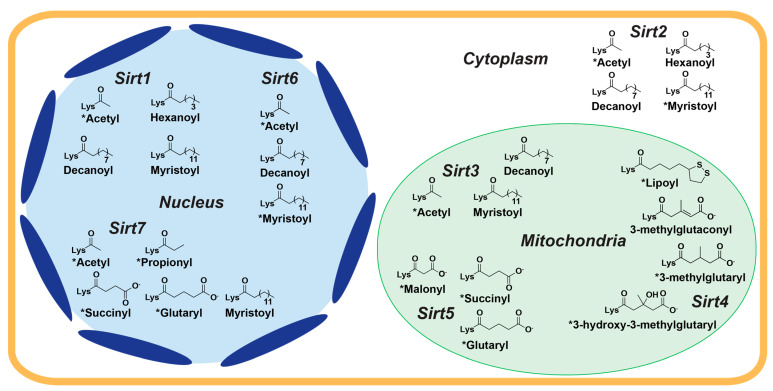
Primary cellular localization and acyl-lysine substrate specificities of human sirtuins. The seven human sirtuin isoforms are differentially distributed between the nucleus (Sirt1, Sirt6, and Sirt7; blue), cytoplasm (Sirt2; white), and mitochondria (Sirt3, Sirt4, and Sirt5; green) and possess distinct acyl-lysine substrate specificities. * = sirtuin deacylase activity confirmed in cells.

While sirtuins harbor overlapping substrate specificities in vitro, subcellular localization of the seven mammalian sirtuins confers cellular selectivity for their endogenous protein targets [11]. Sirt1, Sirt6, and Sirt7 are primarily nuclear, Sirt2 is mainly cytosolic, and Sirt3, Sirt4, and Sirt5 are predominantly mitochondrial (Figure 1) [10]. However, sirtuin isoform localization can vary. Depending on specific cellular stimuli, Sirt1 and Sirt2 can shuttle between the nucleus and cytosol [12,13]. Although contested [14,15], cellular stress has been reported to cause a long unprocessed isoform of Sirt3 to localize to the nucleus [15,16,17]. Sirt5 has also been observed to regulate levels of protein malonylation in both the mitochondria and cytosol [18].

**Figure 2:**
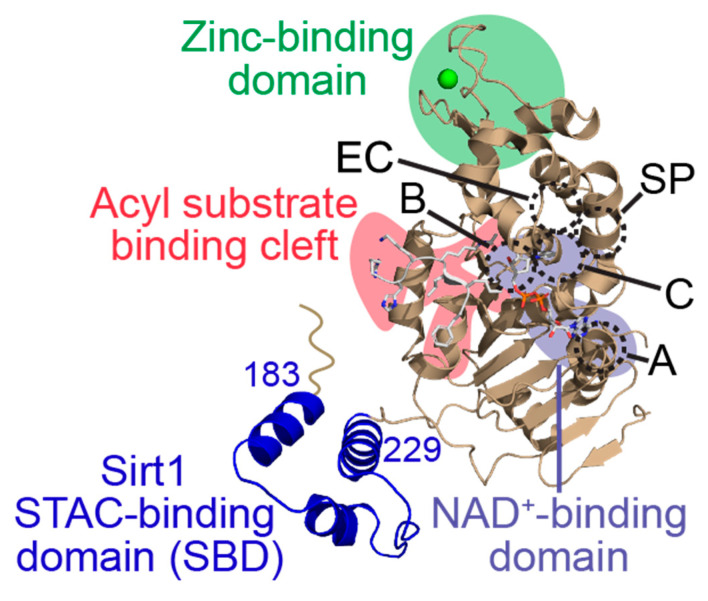
General structure of a human sirtuin deacylase bound to an acylated substrate and co-substrate NAD^+^. Key structural domains include the acylated substrate binding cleft (red), the NAD^+^ co-substrate binding pocket (purple), and the zinc-tetrathiolate domain (green). Within and proximal to the NAD^+^ binding pocket, subregions involved in binding the adenine moiety (A-site), the nicotinamide ribose moiety (B-site), and the nicotinamide ribose moiety upon acyl-substrate binding (C-site) of NAD^+^, as well as the extended C-site (EC) and selectivity pocket (SP) exploited by some sirtuin modulators, are outlined by dashed circles and annotated with the corresponding letters. The sirtuin-activating compound (STAC)-binding domain (SBD; blue) is unique to Sirt1. PDB ID: 4ZZJ [39].

Sirtuins share an evolutionarily conserved ~275 amino acid catalytic core composed of two domains connected by multiple loops: a larger, highly conserved Rossmann fold domain that binds the sirtuin co-substrate NAD^+^ and a smaller, more variable zinc-binding domain; the acylated substrate binds in a cleft formed by these two domains (Figure 2) [19,20]. Variation in the acylated substrate binding cleft allows for differential substrate preferences and deacylase activities [21,22]. Sirtuins remove a variety of acyl modifications from lysine residues (Figure 1) [23]; Sirt1-3 are strong deacetylases [24] but can also remove propionyl [25], butyryl [25], β-hydroxybutyryl [26], crotonyl [27], and long-chain fatty acid (LFA) acyl groups [28]. Sirt5 prefers negatively charged acylations such as malonyl, succinyl, and glutaryl groups [22,29]. Sirt4 removes more complex negatively-charged acylations, such as methylglutaryl, hydroxymethylglutaryl, and methylglutaconyl acyl groups [30] as well as lipoyl and biotinyl acyl groups [31]. Although Sirt6 and Sirt7 are weak deacetylases in vitro [32], their enzymatic activities are stimulated by cellular components. Sirt6 efficiently deacylates LFAs [33], and Sirt6 deacetylase activity is stimulated by biologically relevant free fatty acids (FFAs) [28] and by binding to nucleosomes and nucleosomal DNA [34,35]. Similarly, Sirt7 deacetylase activity is stimulated by nucleosome binding and several types of nucleic acids [36,37,38], while its defatty-acylase activity is primarily stimulated by rRNA [38].

### 1.2. Differential Modulation of Sirtuin Activity

**Figure 3:**
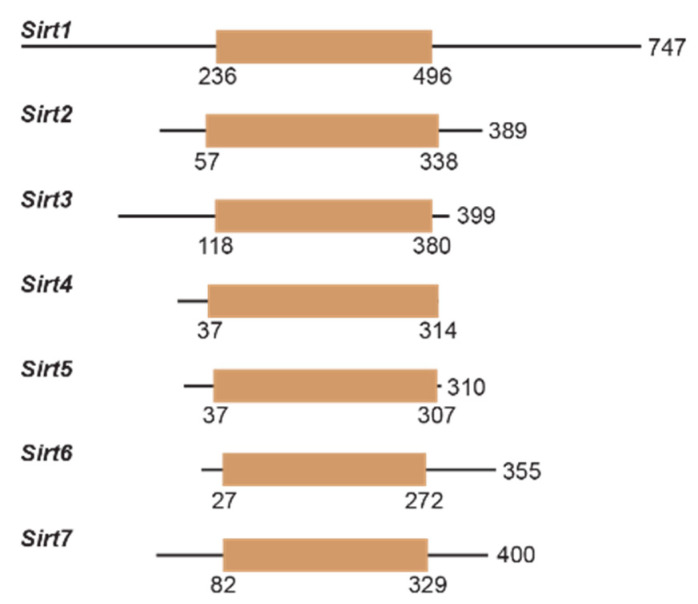
Human sirtuins have variable *N*- and *C*-termini and catalytic cores. Linear representation of Sirt1-7, with tan denoting the catalytic core. Boundaries of the catalytic core were determined from UniProt for each sirtuin isoform.

Sirtuin activity is regulated transcriptionally and by protein–protein interactions, subcellular localization, and nutrient/cofactor availability [32,40,41,42]. In addition to their conserved catalytic core, sirtuins have *N*- and *C*-termini that vary in length (Figure 3). Notably, the lengthy *N*- and *C*-termini of Sirt1 have been found to enhance Sirt1 deacetylase activity [43]. Post-translational modifications, including modifications by oxidants upon cellular stress [44], can also affect sirtuin deacylase activity [45]. Sirtuin attenuation and upregulation have simultaneously been implicated in the pathogenesis of many aging-related diseases, including cancer [46], cardiovascular disease [47], and neurodegeneration [48]. Therefore, small molecule modulators of sirtuin activity have great potential for treating the pathophysiology of aging and extending human lifespans.

Initial efforts to develop sirtuin activators and inhibitors are encouraging. Sirtuin-activating compounds (STACs) have been identified from natural products and optimized with medicinal chemistry approaches; competitive small molecule inhibitors and potent mechanism-based peptide inhibitors have also been developed for most sirtuin isoforms [49,50,51,52]. However, these molecular modulators of sirtuin activity have drawbacks. Many STACs and inhibitors lack cellular activity or selectivity, affecting a variety of other proteins in addition to their intended sirtuin targets [52,53]. Therefore, developing activators and inhibitors that modulate sirtuin activity with greater specificity and cellular efficacy is imperative to clarify the complex roles of sirtuins in the biology of aging and related pathophysiologies. Note that the sirtuin modulators discussed in this review are referred to by the names found in the primary literature.

## 2. Sirtuin Activators

Since the discovery of sirtuin-dependent lifespan extension in yeast [54,55] and mice [56,57], a variety of natural product and synthetic small molecule sirtuin activators have been identified in attempts to extend human lifespan and mitigate aging-related diseases [49,50] (Table 1). Alternative approaches to enhance sirtuin activity are also discussed. Individual or combinatorial strategies to enhance sirtuin activity hold promise for delaying aging and attenuating the pathogenesis of multiple aging-related diseases.

### 2.1. Natural Product Sirtuin Activators

Several natural products activate sirtuins [58]. For example, resveratrol (Table 1), a polyphenol found in grapes and wine [58,68], is a reported Sirt1 activator [58], although these observations have been contested [69,70]. Honokiol (Table 1), a biphenolic molecule found in the bark of magnolia trees, is an alleged Sirt3 activator [61]; however, we have been unable to demonstrate direct Sirt3 activation by honokiol in vitro (unpublished data). Consistent with Sirt6 activation by biological FFAs [28], a screen of fatty acids and lipids found that oleoyl-lysophosphatidic acid (LPA) (Table 1) increased Sirt6 deacetylase activity ~48-fold [65]. LPA also attenuates Sirt1 protein expression [71]. Considering that most natural product activators of sirtuins exhibit several limitations, particularly low specificity [52], we focus our assessment of current sirtuin activators on synthetic small molecules.

### 2.2. Synthetic Small Molecule Sirt1 Activators

The abundant caveats identified with using resveratrol as a selective Sirt1 activator [59,69,70,72,73,74,75,76,77] necessitated the development of synthetic Sirt1 STACs with improved potency and pharmacological properties. Initial attempts to generate synthetic Sirt1 STACs involved modifying the 4′-hydroxyl of resveratrol to reduce redox potential [78]. These efforts resulted in resveratrol derivatives with moderately enhanced stability or Sirt1 activation [78]. Resveratrol has also been modified with the cationic/lipophilic triphenylphosphonium (TPP^+^) group for selective mitochondrial localization [79]. However, given that resveratrol inhibits Sirt3 deacetylase and Sirt5 desuccinylase activities [77], even mitochondrially targeted resveratrol is not predicted to be an effective activator of mitochondrial sirtuins.

The next generation of STACs, structurally unrelated to resveratrol, were identified using high-throughput screening with a fluorescent Sirt1 acetyl-lysine substrate deacetylation assay [80]. This yielded ‘hit’ molecules ~10-fold more potent than resveratrol in cellular assays [80]. Sirtris (now part of GlaxoSmithKline; GSK) employed similar high-throughput methods using fluorescence polarization and mass spectrometry; ‘hit’ molecules from this screen were up to 1000-fold more potent than resveratrol [59,81,82]. Although Sirt1-3 are closely related sirtuin isoforms [83], optimized Sirtris Sirt1 STACs SRT1460, SRT1720 (Table 1), and SRT2183 displayed up to 833- and 1875-fold selectivity for Sirt1 over Sirt2/3 in vitro, and demonstrated a Sirt1-dependent decrease in p53 acetylation in the U2OS osteosarcoma cell line [59]. Kinetic analysis revealed that Sirtris compounds increased Sirt1 deacetylase activity by lowering the Michaelis constant (*K*_M_) of Sirt1 for acetyl-lysine substrates without affecting the *K*_M_ for NAD^+^ [59]. SRT1720 also inhibits Sirt3 by competing with the acyl-lysine substrate for binding [84], suggesting that the mechanism of Sirt1 activation by SRT1720 is unique.

Despite debates regarding the legitimacy of direct STAC-mediated Sirt1 activation [69,70,85], several studies have used biophysical and structural approaches to deconvolute some of the controversies [39,86,87]. STAC activation of Sirt1 depends on aromatic rings in its substrates [86] from fluorophores or natural aromatic amino acids at specific positions relative to the acylated lysine residue [87]. Consistent with this mechanism, STAC-mediated Sirt1 activation was decreased through the mutation of the aromatic amino acids’ *C*-terminal to the acetyl-lysine residue in acetylated Sirt1 peptide substrate proliferator-activated receptor γ coactivator 1α (PGC1α) and human fork-head box O3a (FOXO3a) [87]. Additionally, *N*-terminal truncations of Sirt1 adjacent to the catalytic core decreased STAC binding to Sirt1 and corresponding Sirt1 activation, indicating the region *N*-terminal to the Sirt1 active site (amino acid residues 1–224) is required for STAC-mediated activation [87]. An E230K point mutation also attenuated STAC-mediated activation of Sirt1 but did not affect baseline Sirt1 enzyme kinetics or subcellular localization [87], further demonstrating that STAC-mediated Sirt1 activation requires the Sirt1 *N*-terminus. Moreover, myoblasts lacking Sirt1 or expressing Sirt1 E230K failed to display markers of STAC-mediated activation [87]. Additional structural analysis using site-directed mutagenesis coupled with hydrogen–deuterium exchange mass spectrometry revealed that Sirt1 contains a unique STAC-binding domain (SBD) *N*-terminal to its catalytic core (amino acid residues 183–229) (Figure 2) [39], consistent with the previously reported structural requirements for STAC-mediated Sirt1 activation [87]. Together, these data suggest that all known Sirt1 activators operate by a direct allosteric activation mechanism that includes binding to the Sirt1 SBD and a Sirt1 substrate preference for hydrophobic and aromatic amino acids *C*-terminal to the acyl-lysine residue [39,86,87].

The initial use of Sirtris Sirt1 STACs in vivo is promising. Oral gavage administration of SRT1720 to diet-induced or genetically obese diabetic mice reduced insulin resistance and improved glucose tolerance [59]. Furthermore, SRT1720 extended lifespan, improved metabolic and inflammatory markers, and delayed the onset of age-related pathologies in adult mice fed a high-fat or standard diet [88,89]. However, the effects of Sirt1 STACs in vivo may be mediated by proteins other than Sirt1. Notably, the diabetes-mitigating effects of SRT1720 in mice may depend on AMP-activated protein kinase (AMPK) activation since SRT1720 can activate AMPK independently of Sirt1 [90]. Moreover, SRT1460, SRT1720, and SRT2183 have all demonstrated off-target interactions with numerous receptors, enzymes, transporters, and ion channels [85].

Nonetheless, Sirtris Sirt1 STACs have entered clinical trials to assess pharmacokinetics, tolerability, and efficacy in age-related pathologies. Based on pharmacokinetic properties in animal studies, SRT2104 (Table 1) was the first Sirtris Sirt1 STAC deemed to have translational clinical potential [60]. In phase I clinical trials, SRT2104 was well tolerated by healthy volunteers but exhibited poor oral bioavailability [91]. Consistent with Sirt1 activation and despite its low bioavailability, month-long administration of SRT2104 in elderly patients decreased serum cholesterol and triglyceride levels and increased the HDL/LDL ratio [92]. SRT2104 has also been clinically investigated in cardiovascular function [93], type 2 diabetes [94], and other inflammatory diseases [95,96,97]. Due to the poor bioavailability of SRT2104 [91], these subsequent clinical trials exhibited inter-subject variability in drug exposure and mixed outcomes [93,94,96,97]. Accordingly, once their pharmacological properties are optimized, SRT2104 and analogous Sirt1 STACs will likely demonstrate greater therapeutic benefits for age-related pathologies.

### 2.3. Synthetic Small Molecule Sirt3 and Sirt5 Activators

While Sirt1 small molecule activators have been widely reported, small molecule activators of other sirtuin isoforms are slowly emerging. High-throughput screening and structure-based design generated ADTL-SA1215 (Table 1), an allosteric Sirt3 activator with high selectivity over Sirt2/5 and moderate selectivity over Sirt1 in vitro [62]. Selectivity is likely conferred by the unique interaction of ADTL-SA1215 with Sirt3 via a hydrophobic allosteric pocket near the Sirt3 acyl-lysine binding site [62]. ADTL-SA1215 exhibited Sirt3-dependent cellular activity, increasing Sirt3 deacetylase activity ~two-fold and decreasing acetylation of the Sirt3 substrate manganese superoxide dismutase (MnSOD) at lysine-68 and -122 in the MDA-MB-231 triple-negative breast cancer cell line in a concentration-dependent manner [62]. ADTL-SA1215 also induced autophagy of MDA-MB-231 cells both in vitro and in vivo in a murine xenograft model of triple-negative breast cancer via Sirt3-mediated regulation of multiple autophagy-related proteins [62], confirming the efficacy of Sirt3 activation in this cancer model.

1,4-dihydropyridines [98,99], first reported as weak Sirt1-3 activators [98], have recently been optimized with structure–activity relationship (SAR) studies to develop next-generation Sirt3 activators [63]. Compound **31**, the best identified Sirt3 activator, increased Sirt3 deacetylase activity up to ~1000-fold and exhibited strong Sirt3 selectivity in vitro, with minimal impacts on Sirt1/2/5/6 and only partial inhibition of Sirt4 at 100 μM [63]. Consistent with cellular Sirt3 activation, Compound **31** decreased acetylation of the Sirt3 target glutamate dehydrogenase in MDA-MB-231 cells [63]. Further SAR studies based on Compound **31** have yielded additional 1,4-dihydropyridine-based Sirt3 activators with demonstrated capacity to decrease viability and colony formation in MDA-MB-231 cells and the CAL-62 thyroid cancer cell line [100].

The same SAR studies that generated 1,4-dihydropyridine Compound **31** also developed Compound **30** as a Sirt5 activator [63]. Compound **30** increased Sirt5 desuccinylase activity up to ~5 fold and maintained Sirt5 selectivity in vitro, showing little effect on Sirt1-3 and only partial inhibition of Sirt4/6 at 100 μM [63]. Decreased succinylation of the Sirt5 substrates pyruvate dehydrogenase subunit A1 (PDHA1) derived from porcine heart tissue and glutaminase in MDA-MB-231 cells confirmed that Compound **30** is cell active [63]. An additional 1,4-dihydropyridine Sirt5 activator, MC3138, did not impact Sirt1/3 deacetylase activity at a concentration of 100 μM in vitro and demonstrated on-target cellular Sirt5 activation via decreased acylation of the Sirt5 substrate glutamate oxaloacetate transaminase 1 (GOT1) in pancreatic cancer cell lines [101]. Notably, MC3138 reduced tumor size in a mouse xenograft model of pancreatic cancer alone and in combination with gemcitabine with no significant side effects [101]. Despite the novelty of these new 1,4-dihydropyridine Sirt3 activators, the lack of animal model testing demonstrates the current superiority of ADTL-SA1215 as the Sirt3 activator of choice for probing the roles of Sirt3 in cancer. However, the promising in vitro and in vivo data derived from 1,4-dihydropyridine Sirt5 activators highlights the need for further studies to enhance their solubility and potency.

### 2.4. Synthetic Small Molecule Sirt6 Activators

To further the development of non-Sirt1 STACs, in silico screening of a small molecule library against Sirt2/3/5/6, coupled with hit testing in a fluorescent deacetylation assay, identified a pyrrolo[1,2-*a*]quinoxaline compound that weakly stimulated Sirt6 deacetylase activity [102]. Derivatization of this parent compound yielded UBCS039 (Table 1), which increased Sirt6 deacetylase activity ~2–3.5 fold without significantly impacting Sirt1-3 deacetylase activity in vitro [64]. Such selectivity is likely derived from the binding of UBCS039 to the hydrophobic portion of the extended Sirt6 acylated substrate binding cleft that facilitates binding to longer acylated substrate chains or fatty acid activators [28,64]. Consistent with a mechanism of Sirt6 activation, UBCS039 increased deacetylation of the Sirt6 substrates histone H3K9 and H3K18 in vitro [64]. Cellular activity of UBCS039 was demonstrated by decreased H3K9 acetylation and Sirt6-mediated increases in autophagosome formation and autophagy-induced cell death in several cancer cell lines [103]. However, as UBCS039 also activates Sirt5 desuccinylase activity by ~two-fold in vitro [64], further optimization is required to enhance UBCS039 sirtuin isoform specificity before it can serve as an effective Sirt6 activator and biological probe. Recent SAR studies based on UBCS039 yielded pyrrolo[1,2-*a*]quinoxaline analogs that increase Sirt6 deacetylase activity up to ~seven-fold, retain selectivity for Sirt6 over Sirt1-3, and do not impact Sirt5 desuccinylase activity [104].

Allostery prediction, virtual library screening, and optimization of a phenylsulfonamide scaffold have also yielded MDL-800 and MDL-801 as Sirt6 activators [105]. Both compounds increased Sirt6 deacetylase activity up to 22-fold and did not modulate Sirt6 demyristoylase or ADP-ribosyltransferase activities [105]. In addition, neither compound had any effects on Sirt1/3/4 and only minimal impacts on Sirt2/5/7, requiring concentrations at least 17 times higher to induce activation [105]. Modeling demonstrated that MDL-800 and MDL-801 activate Sirt6 by allosterically binding to a hydrophobic *N*-terminal pocket defined by the *N*-terminal residues 1–7, V70, E74, F82, F86, V153, and M157 [105]. Notably, MDL-800 decreased acetylation of the Sirt6 substrates histone H3K9 and H3K56 in human hepatocellular carcinoma (HCC) and non-small cell lung cancer (NSCLC) cell lines in a concentration-dependent manner and induced Sirt6-mediated G_0_–G_1_ phase cell cycle arrest [105,106]. Furthermore, MDL-800 decreased the growth of HCC and NSCLC xenografts in mice [105,106]. SAR studies based on MDL-800 and MDL-801 have yielded analog MDL-811 (Table 1), which activates Sirt6 deacetylase activity two times better than MDL-800 and retains selectivity for Sirt6 over Sirt1/2/3/5/7 in vitro [107]. MDL-811 also exhibits potent cellular activity, decreasing tumor formation in a spontaneous mouse model of colorectal cancer [107] and attenuating lipopolysaccharide-induced neuroinflammation and stroke-like brain damage in mice [66].

Additional virtual screening and SAR analyses based on a quinoline scaffold resulted in Sirt6 activator Compound **12q** (Table 1) [67]. Exhibiting nearly 300-fold selectivity for Sirt6 over Sirt1/2/3/5, Compound **12q** activated Sirt6 deacetylase activity two times better than MDL-800 and uniquely stimulated Sirt6 demyristoylase activity up to 15 fold [67]. Compound **12q** cellular activity was confirmed with a concentration-dependent decrease in acetylation of the Sirt6 substrate histone H3K9 in pancreatic cancer cell lines [67]. Compound **12q** also decreased the growth of pancreatic cancer xenografts in mice [67]. The in vivo efficacy of the MDL compound family and Compound **12q** demonstrate that selective Sirt6 activation has therapeutic potential in multiple types of cancer and neurological damage.

### 2.5. Alternative Mechanisms of Sirtuin Activation

**Figure 4:**
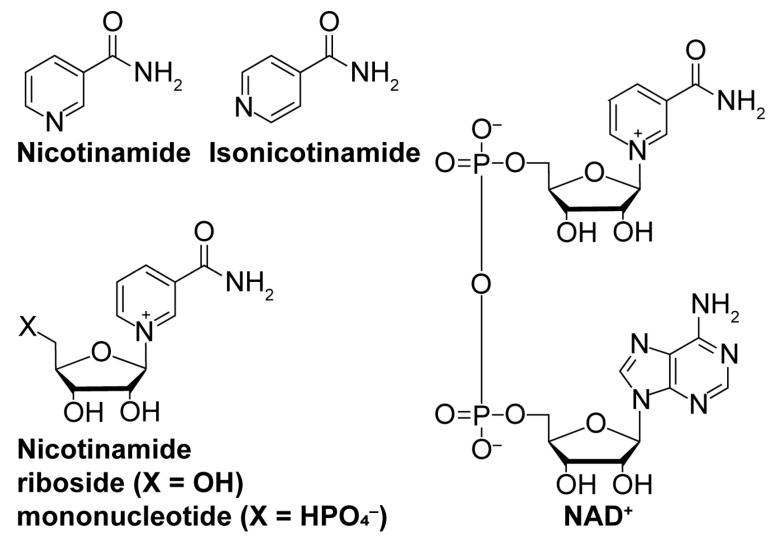
Nicotinamide biomolecules are endogenous regulators of sirtuin deacylation. Structures of key nicotinamide-based biomolecules implicated in sirtuin activity.

While sirtuin activation strategies primarily focus on direct molecular modulation of sirtuin–substrate binding, alternative mechanisms to enhance sirtuin activity exist. Depleting the sirtuin co-substrate NAD^+^ (Figure 4) correlates with the aging-related attenuation of sirtuin activity [108,109]. Therefore, boosting intracellular NAD^+^ levels through supplementation of more cell-permeable NAD^+^ precursors like nicotinamide riboside and nicotinamide mononucleotide (Figure 4) [110,111] or via inhibition of NAD^+^-consuming enzymes (i.e., poly-ADP-ribose polymerases (PARPs) [112] and NADase CD38 [113]) could fortify sirtuin activity to lessen aging-related pathologies. Additionally, sirtuins are autoinhibited by nicotinamide (NAM; Figure 4), a product of sirtuin-catalyzed deacylation and a noncompetitive inhibitor of sirtuin deacylase activity [114]. Therefore, relieving NAM autoinhibition with isonicotinamide (Figure 4), an NAM isostere that competes with NAM for binding to sirtuins, could have similar restorative effects on sirtuin activity [115].

Sirtuins can also be modulated by post-translational modifications, including phosphorylation, methylation, and sumoylation [45]. In particular, sirtuin activity can be inhibited by post-translational modifications resulting from cellular oxidative stress [44,116]. For example, sirtuins are differentially sensitive to inhibition by cysteine *S*-nitrosation, glutathionylation, and sulfenylation [116]. We recently reviewed the effects of oxidative post-translational modifications on sirtuin structure and function [44]. Accordingly, using specific oxidant scavengers corresponding to the inhibitory oxidative modification, inhibitors of oxidant-producing enzymes (e.g., nitric oxide synthase inhibitors), or using antioxidants more generally could indirectly restore sirtuin activity during oxidative stress.

The use of epigenetic small molecules to increase sirtuin transcription and protein expression provide another potential mechanism for sirtuin activation [117,118,119]. Indeed, selective inhibition of the bromodomain and extraterminal domain (BET) protein family by BET bromodomain inhibitors (e.g., JQ1, I-BET151, and I-BET762) elevates Sirt1 transcript and protein levels in a variety of cell lines (unpublished data) [117,118,119]. BET bromodomain inhibition by JQ1 also increases Sirt1-dependent p53 deacetylation without directly regulating Sirt1 deacetylase activity [117]. However, BET bromodomain inhibitors decrease the protein expression of Sirt1 and other sirtuins in additional cell lines [118,119] and broadly impact gene expression across many transcriptional networks [120]; thus, studies with more selective BET bromodomain inhibitors are required to fully elucidate the impact of BET inhibition on sirtuin protein expression and reduce off-target epigenetic effects.

## 3. Small Molecule Sirtuin Inhibitors

A large class of sirtuin inhibitors consists of small molecules (Table 2) that can be distinguished by their hypothesized or structurally determined sirtuin binding sites, as well as their kinetics of inhibition relative to sirtuin acylated substrates and/or NAD^+^. Extensive studies based on computational modeling, structural biology approaches, and biochemical data delineate the specific binding sites and inhibition mechanisms of these compounds [121,122,123,124,125]. Such studies inform improvements in the overall potency and isoform selectivity of existing small molecule inhibitors and facilitate testing of inhibitors with high translational potential in aging-related diseases.

### 3.1. Small Molecule Sirtuin Inhibitors Targeting the Acylated Substrate Binding Site

Competitive inhibition by small molecules at the sirtuin acylated substrate binding cleft increases the apparent *K*_M_ for acyl-lysine substrates, which lowers the sirtuin deacylase activity when substrate concentrations are not saturating. Hydroxynaphthaldehydes (e.g., sirtinol, salermide, splitomicin, and cambinol) comprise a large class of sirtuin inhibitors that impact the sirtuin catalytic mechanism in this manner with modest potency (average half-maximal inhibitory concentration (IC_50_) of ~64 μM) [1,134,135,136] and reported apoptotic activity in cancer cell lines [134,136]. However, the selectivity of hydroxynaphthaldehydes for specific sirtuin isoforms was not rigorously tested [134,136]. The better-characterized cambinol analogs have been optimized into compounds that selectively inhibit Sirt1 (IC_50_ = 27 μM) with >7-fold selectivity for Sirt1 over Sirt2/3, Sirt2 (IC_50_ = 13 μM) with >15-fold selectivity for Sirt2 over Sirt1/3, or Sirt3 (IC_50_ = 6 μM) with ~5–7-fold selectivity for Sirt3 over Sirt1/2 [137]. Increased acetylation of Sirt1 substrate p53 and Sirt2 substrate α-tubulin demonstrated that selective Sirt1 and Sirt2 inhibitors possessed on-target cellular activity in the NCI-H460 NSCLC cell line [137]. Additional SAR studies of cambinol have generated open-ring cambinol analogs that selectively inhibit Sirt2 with nanomolar potency and moderate selectivity for Sirt2 over Sirt1/3 [138]. These cambinol analogs exhibited on-target Sirt2 inhibition in the NCI-H60 NSCLC cell line, as demonstrated by a concentration-dependent increase in α-tubulin acetylation [138]. The cambinol analogs also induced cytotoxicity in a panel of cancer cell lines [138].

A virtual screen of over 25 million compounds identified ICL-SIRT078 as a Sirt2 inhibitor with an IC_50_ value of 1.45 μM and >50-fold selectivity for Sirt2 over Sirt1/3/5 in vitro [121]. Molecular modeling suggested that the high selectivity of ICL-SIRT078 for Sirt2 over other sirtuins is conferred by a strong network of hydrophobic interactions with the Sirt2 acylated substrate binding cleft, which are disrupted by loops present in Sirt1/3/5[121]. On-target Sirt2 inhibition by ICL-SIRT078 was observed in the MCF7 breast cancer cell line via increased acetylation of the Sirt2 substrate α-tubulin [121]. ICL-SIRT078 treatment of a rat dopaminergic neural cell line also attenuated loss of cell viability in induced Parkinson’s disease cell death [121], consistent with prior reports of the benefits of Sirt2 inhibition in Parkinson’s disease [139].

Virtual library screening against a Sirt4 homology model, coupled with SAR optimization of sulfur dioxide-containing polycyclic hits, recently resulted in the first reported Sirt4 inhibitors [132]. The most potent inhibitor, Compound **60** (Table 2), inhibited Sirt4 β-hydroxy β-methylglutarylation (de-HMGylation) with an IC_50_ value of 0.9 μM [132]. Although 10 μM of Compound **60** was sufficient to inhibit Sirt2 and Sirt4, Compound **60** maintained ~3.5–5.5-fold selectivity for Sirt4 over Sirt1/3/5/6 at 10 μM [132]. In contrast, less-potent Compound **69** (Table 2) (IC_50_ = 16 μM) exhibited ~two–three-fold selectivity for Sirt4 over Sirt1/2/3/5/6 at 50 μM [132]. Computational docking predicted that the compounds bind in the Sirt4 acylated substrate binding cleft; follow-up kinetic studies with Compound **69** suggest that the inhibitor is competitive with respect to the acylated substrate [132]. Sirt4 decreases pyruvate dehydrogenase complex activity via delipoylation of dihydrolipoyllysine acetyltransferase [31]. Consistent with on-target cellular Sirt4 inhibition, Compounds **60** and **69** concentration-dependently increased pyruvate dehydrogenase complex activity in C2C12 mouse myoblast cells pretreated with the pyruvate dehydrogenase complex inhibitor Glutamax [132]. These first-in-class Sirt4 inhibitors provide attractive scaffolds for further development of Sirt4 inhibitors with increased potency and sirtuin isoform specificity [132].

Sirt5 is also a recent target of sirtuin inhibitor development [133,140,141]. Since glutarylation is a Sirt5 acylation substrate [29], 3-thioureidopropanoic acid derivatives were developed to competitively inhibit Sirt5 by imitating glutarylated Sirt5 substrates [141]. The most potent derivatives inhibited Sirt5 desuccinylase activity with an IC_50_ value of ~3–4 μM and exhibited ~150–200-fold selectivity for Sirt5 over Sirt1/2/3/6 [141]. Computational docking predicted that the compounds bind in the Sirt5 acylated substrate binding cleft; kinetic assays supported these predictions by demonstrating competitive inhibition against a fluorogenic Sirt5 succinylated substrate [141]. Further SAR studies yielded Compound **58**, an acylated substrate-competitive Sirt5 inhibitor with nanomolar potency (IC_50_ = 310 nM) and improved selectivity for Sirt5 over Sirt1/3 [140]. Notably, Compound **58** improved kidney function in two mouse models of sepsis-induced acute kidney injury, though the mechanism by which Sirt5 inhibition exerts kidney-protective effects is unknown [140]. Screening a 5000-member drug-like compound library coupled with SAR optimization of the pyrazolone-containing hit also produced Sirt5 inhibitor Compound **47** (Table 2), with an IC_50_ value of 210 nM and >3800-fold selectivity for Sirt5 over Sirt1/2/3/6 [133]. Like the other reported Sirt5 inhibitors [140,141], Compound **47** was predicted to bind the Sirt5 acylated substrate binding pocket and exhibited competitive inhibition against a Sirt5 succinylated substrate [133].

Taken together, small molecules competitively targeting the sirtuin acyl-lysine binding site pose a rational mechanism for inhibiting sirtuin deacylase activity. However, with the potential exception of ICL-SIRT078 (Sirt2) and Compound **47** (Sirt5), most inhibitors targeting the sirtuin acylated substrate binding site require optimization to enhance sirtuin isoform selectivity. Moreover, every member of this inhibitor class would benefit from studies to increase the overall potency and confirm the mechanistic efficacy of sirtuin inhibition within in vivo models of cancer and neurodegeneration.

### 3.2. Small Molecule Sirtuin Inhibitors Targeting the NAD^+^ Binding Site

Small molecule inhibitors interacting with the sirtuin NAD^+^ binding site increase the apparent *K*_M_ for the NAD^+^ co-substrate, providing an additional mode of sirtuin inhibition. Several small molecule sirtuin inhibitors targeting the NAD^+^ binding site have been identified through various high-throughput screens [127,129,130]. Elixir Pharmaceuticals combined this approach with SAR studies to develop EX-527 (selisistat) (Table 2), which potently inhibited Sirt1 with an IC_50_ value of 38–98 nM in vitro [127,142]. Moreover, EX-527 demonstrated 200-fold and ~500-fold selectivity for Sirt1 over Sirt2 and Sirt3, respectively [127]. EX-527 exhibited on-target Sirt1 inhibition in multiple cell lines exposed to DNA-damaging agents, demonstrated by increased acetylation of the Sirt1 substrate p53 [142]. The kinetics of in vitro Sirt1 deacetylation assays suggested that EX-527 is an uncompetitive inhibitor with respect to NAD^+^ and likely binds to the Sirt1-substrate complex after nicotinamide release from the Sirt1 active site [127]. This proposed mechanism of EX-527-mediated Sirt1 inhibition was confirmed using crystallography with Sirt1 bacterial homolog Sir2Tm and human Sirt3, where the active *S*-enantiomer of EX-527 (EX-243) [142] bound both the C-site (the portion of the sirtuin NAD^+^ binding pocket involved in the cleavage of the NAD^+^ nicotinamide ribosyl bond [143]; Figure 2) and a unique hydrophobic pocket adjacent to the C-site (the extended C-site; Figure 2) and directly interact with the *O*AADPr sirtuin catalysis product [122]. However, since EX-527 binds and inhibits Sirt2/3 at micromolar concentrations [122,127], the selectivity of EX-527 for Sirt1 is likely attributed to the differential kinetics of the enzyme–product–inhibitor complex formation and subsequent product release rather than specific Sirt1 structural features [122].

Enthusiasm for the clinical potential of EX-527 in neurodegenerative diseases was sparked in part by a study demonstrating that EX-527 attenuated functional motor deficits and decreased striatal huntingtin protein inclusions in a mouse model of Huntington’s disease [144]; however, a phase II clinical trial demonstrated that EX-527, while a pharmacologically safe drug, did not improve Huntington’s disease patient outcomes within the timeframe of the study [145]. Nonetheless, the search for alternative therapeutic applications of EX-527 is ongoing, as highlighted by a recent study demonstrating that EX-527 attenuates diabetic nephropathy in a rat model of type 2 diabetes [146] and by the recently ended phase I clinical trial exploring the benefits of EX-527-mediated Sirt1 inhibition for infertility (NCT04184323).

Because commonly tested cellular concentrations of EX-527 are sufficient to inhibit other human sirtuins such as Sirt2 [147,148,149,150], small molecule Sirt1 inhibitor isoform selectivity can still be improved. To this end, a high-throughput luciferase deacetylation assay for Sirt1 inhibitors, coupled with SAR optimization of the benzoxazine scaffold hit, generated Compound **4.27**, which inhibited Sirt1 with an IC_50_ value of 110 nM and slightly improved selectivity for Sirt1 relative to EX-527 [147]. MDA-MB-231 breast cancer cells treated with Compound **4.27** after etoposide pre-treatment exhibited increased p53 acetylation, consistent with Compound **4.27** exhibiting on-target cellular inhibition of Sirt1 [147]. Like EX-527, kinetic analyses revealed that Compound **4.27** is an uncompetitive inhibitor of Sirt1 with respect to NAD^+^, and molecular docking predicted that Compound **4.27** binds in part of the Sirt1 extended C-site (Figure 2) [147]. Thus, Compound **4.27** is a promising starting point to develop selective Sirt1 inhibitors that can be tested for therapeutic potential in Huntington’s disease and other aging pathologies.

To identify more potent Sirt2 inhibitors, Compound **B2**, previously found to be a weak Sirt2 inhibitor, was expanded into a library of 200 structural analogs for screening [129]. SAR studies based on the top hit resulted in AGK2 (Table 2), which inhibited Sirt2 deacetylase activity with an IC_50_ value of 3.5 μM and exhibited >14 fold-selectivity for Sirt2 over Sirt1/3 [129]. Moreover, AGK2 exhibited on-target Sirt2 inhibition in the HeLa cell line, as demonstrated by increased acetylation of the Sirt2 substrate α-tubulin [129]. Molecular docking suggested that AGK2 binds within the Sirt2 C-site (Figure 2), providing a potential mechanism for AGK2-mediated Sirt2 inhibition [129]. Notably, AGK2 treatment of a *Drosophila* model of Parkinson’s disease attenuated α-synuclein-mediated toxicity in dorsomedial neurons, implicating Sirt2 as a therapeutic target for inhibition in Parkinson’s disease [129]. AKG2-mediated Sirt2 inhibition also protected against huntingtin-induced toxicity and protein inclusion formation in a primary striatal neuron cell model of Huntington’s disease by decreasing sterol production [151].

Because AGK2 cannot cross the blood–brain barrier to exert its neuroprotective effects in vivo, a yeast-based high-throughput screen coupled with in silico screening was employed to generate the Sirt2-selective brain-permeable inhibitor AK7 (Table 2) [130]. Like AGK2, AK7 exhibited selectivity for Sirt2 over Sirt1/3 [130] and was predicted to interact with the Sirt2 C-site (Figure 2) [152]. AK7 also decreased cholesterol biosynthesis [130] and exhibited neuroprotective effects in cell and mouse models of Huntington’s disease and Parkinson’s disease [130,152,153]. However, AK7-mediated Sirt2 inhibition showed no therapeutic efficacy in mouse models of amyotrophic lateral sclerosis and ischemic stroke [152], indicating that Sirt2 inhibition only mitigates the pathophysiology of specific neurodegenerative diseases.

The in vitro and in vivo activity of nanomolar-potent sirtuin inhibitors targeting the sirtuin NAD^+^ binding site, such as Compound **4.27** (Sirt1), AGK2 (Sirt2), and AK7 (Sirt2), have demonstrated that small molecules targeting the sirtuin C-site and/or extended C-site (Figure 2) are effective sirtuin inhibitors. In particular, these inhibitors have shown promising therapeutic potential for multiple neurodegenerative disorders. Nonetheless, existing inhibitors require further optimization to improve pharmacologic characteristics like brain permeability.

### 3.3. Adenosine Analogs as Small Molecule Sirtuin Inhibitors

Since the sirtuin co-substrate NAD^+^ and the kinase substrate ATP both contain an adenosine moiety, adenosine mimetic kinase inhibitors may also inhibit sirtuins [123]. To this end, a library containing commercially available kinase inhibitors was screened for ability to inhibit Sirt2 in a fluorescent deacetylation assay [123]. Top hits from this screen included several bisindolylmaleimides (BIMs) [123], known ATP-competitive inhibitors of protein kinase C [154]. The best-identified inhibitor, BIM Ro 31-8220, inhibited Sirt2 deacetylase activity with an IC_50_ value of 800 nM and exhibited ~four-fold selectivity for Sirt2 over Sirt1 [123]. Molecular docking demonstrated that the indole ring of Ro 31-8220 binds in the same location as the NAD^+^ adenine ring on Sirt2, and an in vitro inhibition assay suggested that Ro 31-8220 inhibits Sirt2 by competing with NAD^+^ binding [123]. The cellular activity of Ro 31-8220 was confirmed via hyperacetylation of the Sirt2 substrate α-tubulin in the A549 lung adenocarcinoma cell line [123]. Despite the promising results of Ro 31-8220 in vitro, cross-reactivity of adenosine mimetic sirtuin inhibitors with kinases remains a significant concern; therefore, a thorough examination of cellular off-target effects, and kinase counterscreens in particular, will be required for any future sirtuin inhibitors developed by adenosine mimesis.

### 3.4. Bivalent Small Molecule Sirtuin Inhibitors Targeting the Acylated Substrate and NAD^+^ Binding Sites

Small molecule inhibitors that bind both the sirtuin acylated substrate cleft and NAD^+^ co-substrate binding site may exhibit extremely potent sirtuin inhibition by substantially disrupting the kinetic parameters (i.e., increased *K*_M_) of the sirtuin catalytic mechanism. For example, fragment-based screening coupled with SAR optimization generated the (5-benzamidonaphthalen-1/2-yloxy)nicotinamide analog Compound **64** as a potent Sirt2 inhibitor (IC_50_ = 48 nM) with ~249- and ~915-fold selectivity for Sirt2 over Sirt1 and Sirt3 in vitro [155]. Consistent with its predicted binding to both the C-site and the acylated substrate binding cleft of Sirt2 (Figure 2), kinetic assays suggested that Compound **64** exhibits non-competitive inhibition relative to NAD^+^ and competitive inhibition relative to an acetylated peptide substrate [155]. The cellular activity of Compound **64** was confirmed by increased acetylation of the Sirt2 substrate α-tubulin in a concentration- and time-dependent manner in the MCF7 breast cancer cell line [155].

Further SAR optimization of Compound **64** produced 5-((3-amidobenzyl)oxy)nicotinamide analogs that were also predicted to dually bind to the Sirt2 C-site and acylated substrate binding cleft (Figure 2) [128,156]. Many of the new nicotinamide analogs, like Compound **86** (Table 2), retained selectivity for Sirt2 over Sirt1/3 [128,156] and increased potency against Sirt2 up to ~three-fold compared to Compound **64** [128]. Based on prior studies demonstrating the benefits of Sirt2 inhibition in neurodegenerative diseases with inhibitors targeting the Sirt2 NAD^+^ binding site [129,130,151,152,153], two nicotinamide analogs with predicted brain permeability were tested in an α-synuclein toxicity model of Parkinson’s disease in SH-SY5Y neuroblastoma cells [128]. Both nicotinamide analogs attenuated α-synuclein-induced SH-SY5Y viability loss [128], suggesting that Sirt2 inhibitors with dual binding modes retain translational potential in neurodegenerative diseases [128].

Aside from nicotinamide analogs, library screening with a fluorescent acetylation assay yielded aminothiazole-based sirtuin-rearranging ligands (i.e., SirReals) as Sirt2 inhibitors [124]. SirReal2 (Table 2), the most potent of these compounds, inhibited Sirt2 with an IC_50_ value of 140 nM and exhibited high (>1000-fold) selectivity for Sirt2 over Sirt1/3/4/5/6 [124]. Sirt2 selectivity is derived from the unique binding mode of SirReal2 [124]. Crystallization demonstrated that SirReal2 not only engages the extended C-site and protrudes into the Sirt2 acylated substrate binding cleft but also triggers structural reorganization of the Sirt2 active site to bind to an additional ‘selectivity pocket’ next to the extended C-site (Figure 2) [124]. SirReal2 exhibited on-target Sirt2 inhibition in HeLa cells, demonstrated by increased acetylation of the Sirt2 substrate α-tubulin and decreased protein levels of the mitotic spindle assembly checkpoint protein BubR1 [124], whose proteasomal degradation is inhibited by Sirt2-mediated deacetylation [157]. SAR optimization generated additional SirReals with improved cellular activity and/or solubility [158,159] and a Sirt2 affinity probe [159]. Thiazoles as a scaffold for Sirt2 inhibition have continued to be investigated; the thiazole MIND4 (Table 2) was found to inhibit Sirt2 with an IC_50_ value of 3.5 μM and independently activate nuclear factor erythroid 2-related factor 2 (NRF2), resulting in synergistic neuroprotection in several models of Huntington’s disease [131].

Although most small molecules targeting the sirtuin NAD^+^ binding site and acylated substrate binding cleft are directed against Sirt2, bivalent sirtuin inhibitors targeting other sirtuin isoforms are appearing. For example, potent (low nM IC_50_) yet non-selective Sirt1-3 inhibitors like ELT-31 (Table 2) have been identified through DNA-encoded library screening coupled with SAR optimization [126]. Analogous to the nanomolar-potent nicotinamide analog Sirt2 inhibitors [128,155], the potent sirtuin inhibition exhibited by the ELT compound family is derived from its simultaneous engagement of the sirtuin C-site and acylated substrate binding cleft (Figure 2) [126]. In addition, Sirt3 inhibitor LC-0296 was developed to target overexpressed Sirt3 in oral squamous cell carcinoma (OSCC) [160]. LC-0296 inhibited Sirt3 with an IC_50_ value of 3.6 μM in vitro and exhibited ~19- and ~9-fold selectivity for Sirt3 over Sirt1 and Sirt2, respectively [160]. Though the mechanism by which LC-0296 binds and inhibits Sirt3 has not been determined, the polycyclic LC-0296 may interact with both the Sirt3 acyl-lysine and NAD^+^ binding sites via moieties resembling acetyl-lysine and nicotinamide [160]. Consistent with mitochondrial Sirt3 inhibition, LC-0296 increased global mitochondrial protein acetylation, acetylation of the Sirt3 target glutamate dehydrogenase, and reactive oxygen species levels in head and neck squamous cell carcinoma (HNSCC) cell lines [160]. By disrupting the mitochondrial redox balance, LC-0296 promoted HNSCC cell apoptosis and enhanced HNSCC sensitivity to radiation and cisplatin [160].

Surprisingly, the Zn^2+^-chelating class I/II HDAC inhibitor trichostatin A was found to inhibit Sirt6 via a Zn^2+^-independent mechanism [161,162]. Initially hypothesized to chelate the Zn^2+^ in the sirtuin zinc-binding domain (Figure 2) [161], trichostatin A was instead observed to bind the Sirt6 C-site (Figure 2) and the unique Sirt6 extension of the sirtuin acyl-substrate binding cleft [162]. Consistent with this dual binding occupancy, trichostatin A exhibited competitive inhibition relative to the acylated substrate and noncompetitive inhibition relative to NAD^+^ [161]. Trichostatin A inhibited Sirt6-mediated histone H3K9 and p53 K382 deacetylation with an inhibition constant (*K*_i_) of 2–4.6 μM and did not affect Sirt12/3/5 deacylase activity at concentrations below 50 μM [161]. However, given its well-characterized primary inhibition of class I HDACs (HDAC1-3) and class II HDACs (HDAC4, 6, 7, and 9) [163], trichostatin A is not an effective probe for studying Sirt6 cellular functions.

Most bivalent small molecule sirtuin inhibitors (e.g., Compound **64**, SirReal2, and ELT-31) exhibit nanomolar potency towards their specified sirtuin isoforms. Thus, dual inhibition of the sirtuin acylated substrate and NAD^+^ binding is an attractive mechanism for disrupting sirtuin catalytic activity. Therefore, optimizing and diversifying the interactions of bivalent sirtuin inhibitors with the acylated substrate binding cleft and the C-site/extended C-site (Figure 2) are critical next steps for enhancing sirtuin isoform specificity. Moreover, SAR studies of the most potent inhibitors to enhance pharmacological properties like solubility and membrane permeability will be vital in generating therapeutically relevant compounds for in vivo testing in multiple models of aging-related diseases, such as neurodegenerative diseases and cancer.

### 3.5. Allosteric Small Molecule Inhibitors

While most sirtuin inhibitors engage the acylated substrate channel, NAD^+^ binding site, or both, some inhibitors bind to unique allosteric sites on sirtuins. The identification and development of allosteric sirtuin inhibitors has been accelerated by recent advances in the computational tools to predict potential allosteric sites [164]. For example, reversed allostery prediction methods, coupled with site-directed mutagenesis, generated the allosteric Sirt6 inhibitor JYQ-42 [165]. JYQ-42 inhibited Sirt6 deacetylase activity with an IC_50_ value of 2.33 μM [165]. JYQ-42 showed no effect on the deacetylase activity of any other sirtuin except Sirt2, which required a ~37 times greater concentration for inhibition [165]. Molecular modeling suggested that JYQ-42 bound to pocket Z, an allosteric pocket on Sirt6 formed via orthosteric NAD^+^ binding [165]. Mutations of pocket Z residues increased the IC_50_ of Sirt6 inhibition by JYQ-42, and in vitro kinetic assays demonstrated non-competitive inhibition of JYQ-42 relative to both the acylated substrate and NAD^+^, supporting an allosteric binding mechanism [165]. JYQ-42 increased acetylation of the Sirt6 substrates histone H3K9, H3K18, and H3K56 in pancreatic cancer cell lines in a concentration-dependent manner, consistent with on-target Sirt6 inhibition [165]. JYQ-42 also reduced the expression and secretion of the cancer-induced inflammatory mediators IL-6, IL-8, and TNFα [165]. Moreover, JYQ-42 decreased pancreatic cancer cell migration in a time and concentration-dependent manner [165].

Virtual screening of Sirt6 against a library of marine natural products and derivatives, coupled with SAR optimization of a pyrrol-pyridinimidazole-containing hit, produced Compound **8a** as an additional Sirt6 inhibitor (IC_50_ = 7.5 μM) with ~11–12-fold selectivity for Sirt6 over Sirt1/2 and >26-fold selectivity for Sirt6 over Sirt3/5 in vitro [166]. Consistent with predicted binding to Sirt6 outside of the acylated substrate and NAD^+^ binding pockets, Compound **8a** exhibited non-competitive inhibition relative to both the acetylated substrate and NAD^+^ [166]. Treatment of pancreatic cancer cell lines with Compound **8a** increased acetylation of histone H3K9, consistent with cellular activity against Sirt6 [166]. Compound **8a** exhibited synergistic cytotoxic effects with gemcitabine in a mouse xenograft model of pancreatic cancer without impacting overall pancreatic function [166], supporting the potential therapeutic efficacy of Sirt6 inhibition in pancreatic cancer identified in previous studies [165].

Since the discovery of the Sirt2 ‘selectivity pocket’ (Figure 2), additional small molecule sirtuin inhibitors have exploited this and other unique hydrophobic regions generated upon inhibitor binding [125,167,168,169,170,171]. For example, SAR refinement of a *N*-(3-(phenoxymethyl)phenyl)acetamide scaffold yielded compounds that inhibited Sirt2 deacetylase (IC_50_ = 42–850 nM) and dedecanoylase (IC_50_ = 8.3–17.6 μM) activity [168,171] and exhibited seven-fold selectivity for Sirt2 over Sirt1/3 in vitro [168]. Crystallography demonstrated these inhibitors induce and bind the Sirt2 ‘selectivity pocket’ (Figure 2) and directly inhibit the binding of acylated substrates to Sirt2 by rearranging portions of the active site [171]. In addition, screening of natural products identified the cytisine derivative NPD11033 as an inhibitor of Sirt2 deacetylase activity, with an IC_50_ value of 460 nM and >40-fold selectivity for Sirt2 over Sirt1/3 [125]. A crystal structure of the inhibitor-bound Sirt2 catalytic domain demonstrated that NPD11033 induced ‘selectivity pocket’ (Figure 2) formation similar to SirReal2, but uniquely formed a direct interaction with critical active site base residue H187[172] through a hydrogen bond [125]. Like SirReal2 [124], NDP11033 decreased BubR1 protein levels in the PANC-1 pancreatic cancer cell line, consistent with on-target cellular Sirt2 inhibition [125]. NDP11033 and SirReal2 also reduced PANC-1 cell viability, suggesting that targeted Sirt2 inhibition via the ‘selectivity pocket’ (Figure 2) may be beneficial in pancreatic cancer [125].

These studies suggest that sirtuins contain several druggable allosteric sites that can be bound by non-competitive small molecule inhibitors. Without targeting the acylated substrate binding cleft or NAD^+^ binding pocket common to all seven human sirtuins, allosteric inhibitors have the potential to be optimized into compounds that exert superior sirtuin isoform selectivity compared to other small molecule sirtuin inhibitors. However, additional structural studies are required to enhance understanding of the structural requirements for allosteric pocket induction in Sirt2/6 and identify allosteric pockets on the remaining sirtuin isoforms.

## 4. Mechanism-Based Sirtuin Inhibitors

Mechanism-based compounds that stall or reverse sirtuin catalysis via active site chemistry have emerged as another significant class of sirtuin inhibitors (Table 3). Nicotinamide is a well-known non-competitive inhibitor [173] of all sirtuin isoforms [174]. As a result, various nicotinamide analogs, which interact with the sirtuin active site in a manner analogous to nicotinamide, have been developed as sirtuin deacylase inhibitors. Alternative classes of mechanistic sirtuin inhibitors are broadly classified as linear or cyclic peptidic and pseudopeptidic compounds. These inhibitors contain acyl-substrate warheads that mimic native sirtuin acyl-substrates.

### 4.1. Mechanism-Based Sirtuin Inhibition by Nicotinamide

**Figure 5:**
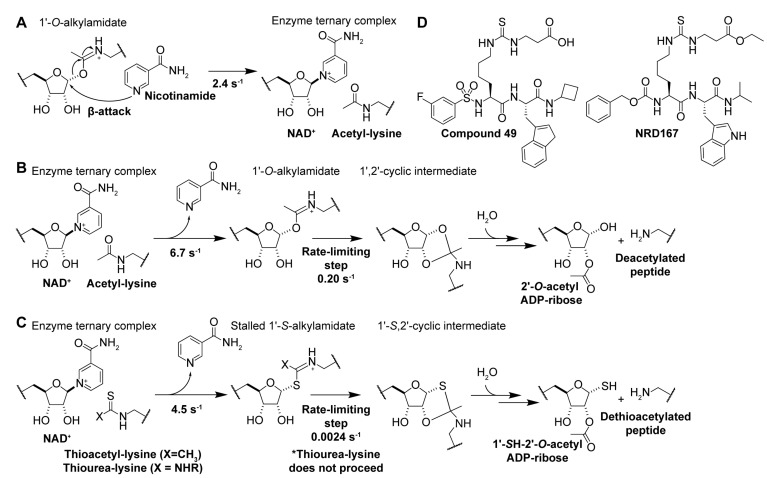
The sirtuin catalytic mechanism provides a rationale for using thioacyl-lysine derivatives as sirtuin inhibitors. (**A**) Mechanism of nicotinamide-mediated sirtuin inhibition. (**B**) Native sirtuin catalytic mechanism with acetylated peptide substrate, yielding 2′-*O*-acetyl-ADP-ribose and the deacetylated peptide substrate. (**C**) Stalled sirtuin catalytic mechanism with thioacetylated and thiourea substrates substituted for the acetylated substrate. (**D**) Thiourea-lysine sirtuin inhibitors. From left to right, Sirt5 inhibitor Compound **49** (a succinyl-thiocarbamoyl pseudopeptide) [201], and Sirt5 inhibitor NRD167 (a succinyl-thiocarbamoyl pseudopeptide) [202].

Rebinding of sirtuin deacylation product nicotinamide [6] to the C-site of the NAD^+^ binding pocket (Figure 2) [174] results in nucleophilic attack of the 1′-*O*-alkylamidate intermediate (Figure 5A), the reverse reaction of the first step in the sirtuin catalytic mechanism (Figure 5B), regenerating NAD^+^ and acyl-lysine substrate [114,174,196]. Accordingly, nicotinamide is a non-competitive inhibitor [173] of all sirtuin isoforms [174] with IC_50_ values ranging from 50–184 µM for Sirt1/2/3/5/6 in vitro [197,198]. In mammals, physiological concentrations of nicotinamide (estimated to be 11–400 μM [199]) fall within this inhibitory range, suggesting that nicotinamide is an endogenous negative regulator of sirtuins [199]. Consistent with this hypothesis, nicotinamide increased acetylation of the Sirt1 substrate p53 in mice [200] and accelerated aging in yeast, comparable to the phenotype of Sir2 knockout [199].

Sirtuin inhibition by nicotinamide exhibits therapeutic effects in several disease models [203,204,205]. Inhibition of Sirt1/2 by nicotinamide was suggested as a mechanism for the decreased cognitive impairment, reduced phospho-tau species, and increased α-tubulin acetylation observed in a transgenic mouse model of Alzheimer’s disease [203]. Nicotinamide also reduced cell viability in prostate and oral cancers through Sirt1 and Sirt3 inhibition, respectively; however, the supra-physiological nicotinamide concentrations’ (5–40 mM) need to observe anti-cancer effects in these studies cast doubt on the therapeutic potential of nicotinamide in such models [204,205]. Notably, nicotinamide is also a precursor for NAD^+^ synthesis [206], which may result in a net stimulation of sirtuin activity due to increased NAD^+^ levels (see Section 2.5).

To circumvent this inherent limitation of nicotinamide-mediated sirtuin inhibition, various nicotinamide analogs have been developed [207,208,209,210]. For example, screening of a nicotinamide and benzamide library identified a 2-anilinobenzamide compound as a modest Sirt1 inhibitor with ~4 and ~14-fold selectivity for Sirt1 over Sirt2 and Sirt3 [207]; cellular activity was confirmed by a concentration-dependent increase in acetylation of the Sirt1 substrate p53 in the HCT116 colorectal cancer cell line [207]. Further SAR studies led to Compound **33a**, which inhibited Sirt2 with an IC_50_ value of 1 μM and exhibited >300-fold selectivity for Sirt2 over Sirt1/3; however, off-target inhibition of class I/II HDACs, CYP3A4, and CYP2D6 was also observed [208]. Cellular Sirt2 inhibition was observed through a concentration-dependent increase in the acetylation of Sirt2 substrate α-tubulin in HCT116 cells without changes in p53 acetylation [208].

Tenovins have also been identified as potential nicotinamide analog sirtuin inhibitors from a cell-based screen of a small molecule library [209]. Consistent with the enhanced p53 protein stability resulting from retention of acetylation [211], tenovins increased p53 protein levels consistent with Sirt1 inhibition and decreased viability of several cancer cell lines [209]. Increased α-tubulin acetylation suggested that tenovins also inhibit Sirt2 [209]. Kinetic studies of tenovin-mediated sirtuin inhibition suggested a non-competitive mechanism towards both the acyl-lysine substrate and NAD^+^ co-substrate [209], analogous to nicotinamide-mediated sirtuin inhibition [6,173]. Further biochemical and biophysical characterization of tenovins has generated derivatives with improved solubility and retention of Sirt1/2-mediated increases in p53 protein and α-tubulin acetylation levels [212] or increases in p21 protein expression [213].

Alternative nicotinamide analogs have been generated through isostere replacement of the nicotinamide amide bond and the introduction of conformational constraints [210]. Compound **2**, a 3-triazolylpyridine (3-TYP) isostere, was the most potent sirtuin inhibitor identified from these syntheses, inhibiting Sirt3 with an IC_50_ value of 38 μM in vitro and exhibiting ~six-fold selectivity for Sirt3 over Sirt1/2 [210]. Treatment of the HeLa cervical cancer cell line and the SK-MEL-28 melanoma cell line with 3-TYP increased mitochondrial protein acetylation and superoxide levels, respectively, consistent with cellular Sirt3 inhibition [210]. However, 3-TYP is also an inhibitor of methionine aminopeptidase 2 (MetAP2) [214] and indoleamine 2,3-dioxygenase 1 (IDO1) [215] and presently is only used as a chemical probe to elucidate Sirt3 biological functions [216]. Therefore, reducing the off-target inhibition of IDO1 and MetAP2 is necessary to develop 3-TYP derivatives with therapeutic potential.

Taken together, nicotinamide and its chemical analogs provide a logical and biologically relevant mechanism of sirtuin deacylase inhibition. However, given the broad-spectrum inhibition of all sirtuin isoforms by nicotinamide [174,197,198], the collateral inhibition of NAD^+^-consuming enzymes PARPs and CD38 (see Section 2.5) by nicotinamide (IC_50_ ~500 μM and *K*_i_ = 920 μM in vitro, respectively [217,218]) and the relatively low sirtuin isoform selectivity of existing nicotinamide analogs, further studies are required to enhance the overall target specificity of nicotinamide analog sirtuin inhibitors. Moreover, improving the selectivity of nicotinamide-based inhibitors for sirtuins over other enzymes that employ nicotinamide as a substrate, like nicotinamide phosphoribosyltransferase of the NAD^+^ salvage pathway (nicotinamide *K*_M_ = 57 nM [219]), will be essential for developing sirtuin inhibitors with translational potential for multiple types of cancer.

### 4.2. Mechanism-Based Sirtuin Inhibition by Thioacetyl-Lysine

Experimental manipulation of the native sirtuin deacylase catalytic pathway (Figure 5B) has demonstrated that substituting the oxygen of the acyl-lysine substrate with sulfur results in potent mechanism-based sirtuin inhibition [175,176,177]. Analogous to the native lysine deacylation mechanism, nucleophilic attack of the nicotinamide ribosyl bond by the thioacyl sulfur (Figure 5C, Table 3) results in rapid nicotinamide production and the formation of a stalled *S*-alkylamidate intermediate [175,176]. Notably, turnover of the *S*-alkylamidate to products 1′-*S*H-2′-*O*-ADP ribose and dethioacylated peptide is ~two orders of magnitude slower than the native deacetylation reaction (Figure 5B vs. Figure 5C) [175,176], resulting in potent mechanism-based inhibition of sirtuin catalysis.

The first thioacetyl-lysine peptide sirtuin inhibitor consisted of a peptide modeled after the *C*-terminus of the Sirt1 substrate p53, with a thioacetyl warhead positioned at a lysine residue near the middle of the peptide sequence (i.e., thioacetyl-p53) [175]. Thioacetyl-p53 inhibited Sirt1 deacetylase activity by decreasing the observed rate constant of deacetylation ~400-fold and did not impact the deacetylase activity of the non-sirtuin HDAC8 [175]. Follow-up studies demonstrated that thioacetyl-p53 exhibited equipotent inhibition of Sirt1/2 (IC_50_ ~2 μM) and ~40-fold selectivity for Sirt1/2 over Sirt3 in vitro [177]. Subsequent thioacetyl peptides modeled after sirtuin isoform-specific substrates α-tubulin and acetyl-CoA synthetase 2 (AceCS2) were developed to target Sirt2 and Sirt3, respectively [177]. Thioacetyl-α-tubulin inhibited Sirt2 with an IC_50_ value of 11.4 μM and showed ~10- and ~40-fold selectivity for Sirt2 over Sirt1 and Sirt3, respectively [177]. Thioacetyl-AceCS2 inhibited Sirt2 and Sirt3 with IC_50_ values of ~4.4 μM [177]; however, the thioacetyl-peptide still exhibited five-fold selectivity for Sirt1 over Sirt2/3, despite being designed to inhibit Sirt3 in vitro [177]. These findings emphasize that Sirt1-3 substrate selectivity, partially governed by varying sirtuin isoform subcellular localization [11], can be superseded by the strong Sirt1-3 sequence conservation [4]. However, truncating a thioacetyl-p53 pentapeptide to a tripeptide resulted in improved inhibition of Sirt1 (IC_50_ = 570 nM) and >250-fold selectivity for Sirt1 over Sirt2 [178]. SAR optimization of non-selective Sirt1-3 thioacetyl-tripeptide inhibitors [179] generated thioacetyl-tripeptides that inhibited Sirt3 with an IC_50_ value of ~1.6 μM with ~9–10-fold selectivity for Sirt3 over Sirt1/2 [180]. Therefore, even minor alterations to a thioacetyl-lysine peptide sequence can significantly change the selectivity of its sirtuin isoform inhibition.

Since the cellular efficacy of peptidic inhibitors is limited by susceptibility to proteolytic degradation and poor membrane permeability [220], non-peptidic and pseudopeptidic scaffolds containing a thioacetyl-lysine warhead have been developed as an alternative class of mechanism-based sirtuin inhibitors [181,182,183]. For example, a Cbz-K(thioacetyl)-NH-Ph scaffold was used to generate Compound **1**, a thioacetyl-lysine pseudopeptide with hydrophobic substituents recognized by amino acid residues at the opening of the sirtuin acylated substrate binding cleft [181]. Compound **1** inhibited Sirt1 with an IC_50_ value of 2.7 μM and demonstrated 8.5 and >300-fold selectivity for Sirt1 over Sirt2 and Sirt3; moreover, a concentration-dependent increase of p53 acetylation in the HCT116 colon cancer cell line suggested that Compound **1** effectively inhibited cellular Sirt1 [181]. Analysis of Sirt1/2 homology models and existing Sirt3 crystal structures, coupled with molecular docking, has also led to Sirt1/2 thioacetyl-lysine pseudopeptide inhibitors with improved potency and cellular activity [182]. In a follow-up study, a fragment-based screening approach was employed to identify optimal *N*- and *C*-terminal pseudopeptide modifications for sirtuin inhibition; the resulting thioacetyl-lysine pseudopeptides effectively inhibited Sirt1-3 (IC_50_ = 0.24–29.4 μM) and increased p53 acetylation in epithelial and neuroblastoma cell lines, indicating on-target cellular inhibition of Sirt1 [183]. Additionally, the optimized pseudopeptides exhibited antiproliferative effects on the A549 lung adenocarcinoma and MCF7 breast cancer cell lines by inducing G_1_ cell cycle arrest [183].

### 4.3. Mechanism-Based Sirtuin Inhibition by Thioacyl-Lysine Derivatives

As an alternative to substrate-specific peptide sequences and truncated peptide lengths, Isoform-selective sirtuin inhibition can also be enhanced by switching the acetyl group of a thioacetyl-lysine warhead to mimic sirtuin-specific acyl substrates [186]. To this end, a thiomyristoyl (TM)-lysine pseudopeptide (Table 3) potently inhibited Sirt2 deacetylation (IC_50_ = 28 nM) with 3500-fold selectivity for Sirt2 over Sirt1 and no significant activity against Sirt3/5/6/7 [186]. Selective TM pseudopeptide-mediated Sirt2 inhibition was confirmed by increased acetylation of the Sirt2 substrate α-tubulin and no changes in the acetylation of the Sirt1 substrate p53 in several breast cancer cell lines [186]. The TM pseudopeptide demonstrated cytotoxic effects in multiple breast cancer cell lines but had less impact on the growth of normal epithelial cell lines, indicating that breast cancer cells are more susceptible to Sirt2 inhibition [186]. Notably, the TM pseudopeptide decreased tumor size or increased overall survival in two mouse models of breast cancer and induced a G_1_/G_0_ cell cycle arrest in MCF7 cells via reduced expression of the oncogene c-Myc, indicating that Sirt2 is an attractive therapeutic target in breast cancer [186]. Subsequent SAR studies generated additional nanomolar-potent TM pseudopeptide inhibitors of Sirt2 deacetylase and demyristoylase activities [188]. Compound **26**, the most potent of these next-generation TM pseudopeptides, exhibited on-target Sirt2 inhibition in MCF7 breast cancer cells, as demonstrated by increased acetylation of perinuclear α-tubulin; Compound **26** also decreased MCF7 cell migration, a proxy for metastatic potential [188].

Glucose conjugation and follow-up SAR studies have also been employed to improve the solubility of TM pseudopeptides [189]. Despite decreased specificity for Sirt2 over Sirt1/3, the optimized TM analogs retained potent inhibition of Sirt2 (IC_50_ = 12–400 nM) [189]. Consistent with ~two-fold higher aqueous solubility relative to the original TM inhibitor, MDA-MB-231 breast cancer cells treated with 100 μM of TM analog NH4-6 (Table 3) demonstrated a ~two-fold greater decrease in viability than cells treated with the original TM pseudopeptide inhibitor at the same concentration [189]. Further SAR studies based on the TM pseudopeptide led to JH-T4, which inhibited Sirt2 deacetylase and demyristoylase activities with IC_50_ values of 30 and 40 nM, respectively [187]. JH-T4 also displayed 10- and 500-fold selectivity for Sirt2 over Sirt1 and Sirt3 in vitro [187]. Additionally, JH-T4 increased cellular fatty acylation of the Sirt2 target K-Ras4a and demonstrated cytotoxicity in multiple cancer cell lines [187]. However, the micromolar concentrations of JH-T4 used in cellular assays also inhibited Sirt1 (IC_50_ = 300 nM), as demonstrated by increased p53 acetylation in MCF7 cells and off-target cytotoxicity in normal epithelial cells treated with JH-T4 [187].

Since JH-T4 also inhibits Sirt3 deacetylase activity (IC_50_ = 2.5–15 μM) at low µM concentrations [187,194], the JH-T4 benzyl carbamoyl moiety was substituted for the cationic/lipophilic TPP^+^ group to enhance Sirt3 inhibition via mitochondrial targeting [194]. The resulting inhibitor YC8-02 demonstrated improved Sirt3 inhibition in vitro and enhanced mitochondrial penetration in the Karpas 422 diffuse large B cell lymphoma (DLBCL) cell line compared to JH-T4; however, YC8-02 still inhibited Sirt1 and Sirt2 with IC_50_ values of 2.8 μM and 62 nM, respectively [194]. YC8-02 increased global mitochondrial protein acetylation and induced metabolic perturbations in Karpas 422 cells; YC8-02 also inhibited tumor growth of DLBCL xenografts in mice [194]. Similar metabolic results were observed with genetic Sirt3 depletion in Karpas 422 cells, suggesting that the effects of YC8-02 in DLBCL models are mediated by Sirt3 inhibition [194]. Additional studies demonstrated that YC8-02 induced metabolic perturbations in leukemic stem cells [221] and increased acetylation of the Sirt3 substrate isocitrate dehydrogenase 2 in MDA-MB-231 cells [222], consistent with its presumed role as a mitochondrially targeted Sirt3 inhibitor.

Since myristoyl-lysine substrates are preferred Sirt6 deacylation targets [33], the TM warhead was integrated into pentapeptide Sirt6 inhibitors based on substrates TNFα or H3K9 [223]. Several pentapeptides inhibited Sirt6 demyristoylase activity (IC_50_ = 1.7–42.2 μM); however, inhibition of Sirt1-3 deacetylase activity was observed at comparable concentrations [223]. Treatment of HEK293T cells with the pentapeptide Sirt6 inhibitors increased TNFα fatty acylation, suggesting that the inhibitors possess on-target cellular activity despite overall low potency and specificity [223].

Analogous to TM, the acyl-lysine substrate specificity of Sirt5 [22] can be mimicked by thiosuccinyl (TS) or thiomalonyl as the warhead in peptide-based Sirt5 inhibitors [197]. Accordingly, a 12-mer peptide with a TS warhead, based on the sequence of Sirt5 substrate H3K9, inhibited Sirt5 desuccinylation with an IC_50_ value of 5 µM and >20-fold selectivity for Sirt5 over Sirt1-3 [197]. In contrast to prior studies with thioacetyl peptide inhibitors [178,180], shortening the length of TS peptides did not improve potency towards Sirt5 [197]. Thioglutaryl pseudopeptides have also been developed as alternative Sirt5 inhibitors with IC_50_ values of 120–730 nM and ~240–5000-fold selectivity for Sirt5 over Sirt1/2/3/6 [224].

Overall, thioacyl analogs represent an attractive scaffold to develop potent and isoform-specific inhibitors that stall the sirtuin catalytic mechanism, especially in cancer. Despite their observed efficacy in vitro and in select preclinical disease models, concerns still exist regarding the safety and durability of thioacyl-lysine sirtuin inhibitors as therapeutic agents. First, thioamides can exert cytotoxic effects via *S*-oxidation [225,226,227]. Furthermore, although thioacyl analogs effectively inhibit sirtuin deacylation, the stalled *S*-alkylamidate is eventually turned over to the dethioacylated substrate and 1′-*SH*-2′-*O*-acyl-ADP-ribose, terminating mechanism-based sirtuin inhibition (Figure 5C) [175,176].

### 4.4. Mechanism-Based Sirtuin Inhibition by Thiourea-Lysine Derivatives

To address the concerns raised by thioacyl analogs [175,176,225,226,227], thiourea analogs represent an ideal alternative; compared to thioamides, thiourea-containing compounds are more easily synthesized [190], exhibit increased cell penetrance [228], are less cytotoxic [225], and are not turned over to sirtuin deacylation products [51,185,190]. Our observation that urea-containing homocitrulline stalls the deacylase activity of yeast sirtuin Hst2 [25] provided the rationale for testing whether thiocarbamoyl-lysine, a thiourea-lysine derivative, could inhibit sirtuins in a mechanism-based manner similar to thioacyl-lysines [185]. Indeed, in the first step of sirtuin catalysis, thiourea-lysine analogs are converted to a stalled *S*-isothiourea intermediate incapable of proceeding further, resulting in persistent mechanism-based sirtuin inhibition (Figure 5B) [51,185,190]. Consistent with this scheme, a peptidomimetic containing thiocarbamoyl-lysine inhibited Sirt1-3 deacetylase activity with IC_50_ values of 57–159 μM, albeit ~13–55-fold less potently than its thioacetyl-lysine counterpart [185]. Using the same tripeptide mimetic scaffold, derivatization of the original thiocarbamoyl modification (-CSNH_2_) to a methyl thiocarbamoyl (-CSNHCH_3_) (Table 3) resulted in a pseudopeptide inhibitor with ~2–26-fold improved potency compared to the parent thiocarbamoyl pseudopeptide inhibitor, inhibiting Sirt1-3 deacetylation with IC_50_ values of 2–50 μM [184]. Notably, the methyl thiocarbamoyl pseudopeptide inhibitor demonstrated ~eight-fold greater antioxidant activity than the analogous thioacetyl pseudopeptide inhibitor when tested at 200 μM in a superoxide radical scavenging assay [184]. The methyl thiocarbamoyl pseudopeptide inhibitor also increased p53 acetylation in the HCT116 colon cancer cell line in a concentration-dependent manner, consistent with on-target cellular activity against Sirt1 [184].

Despite its therapeutic efficacy in breast cancer models [186], the poor aqueous solubility and laborious synthesis of the TM pseudopeptide Sirt2 inhibitor inspired conversion to thiocarbamoyl pseudopeptide analogs with improved solubility and decreased hydrophobicity [190]. Like the TM pseudopeptide, fatty-acyl thiocarbamoyl-lysine analogs AF8, AF10, and AF12, with respective alkyl chains of 7, 9, and 11 carbons (Table 3), inhibited Sirt2 with IC_50_ values of 61–150 nM and exhibited ~180–2500-fold selectivity for Sirt2 over Sirt1/3 [190]; AF8 and AF10 also exhibited superior predicted solubility relative to the TM-lysine pseudopeptide [190]. Consistent with selective Sirt2 inhibition, HCT116 cells co-treated with trichostatin A and AF8 or AF10 exhibited increased acetylation of the Sirt2 substrate α-tubulin without significant increases in acetylation of the Sirt1 substrate p53 [190]. As observed with the TM pseudopeptide inhibitor [186], AF8 and AF10 selectively inhibited the growth of multiple cancer cell lines but not normal epithelial cells [190]. Treatment of HCT116 cells with AF8 or AF10 potently inhibited colony formation and significantly reduced tumor growth in a colon cancer xenograft mouse model [190], suggesting that thiocarbamoyl pseudopeptides may exhibit therapeutic potential for Sirt2 inhibition in cancer.

Myristoyl-thiocarbamoyl pseudopeptides like Compound **17** (Table 3), pan-Sirt1-3 inhibitors with IC_50_ values of 0.4–2 μM in vitro, have been adapted to include mitochondrial-targeting peptide sequences to confer selective cellular inhibition of the primary mitochondrial deacetylase Sirt3 [195]. Treatment of HEK293T cells with Compound **17** did not increase p53 or α-tubulin acetylation, consistent with negligible cellular activities against Sirt1 and Sirt2 [195]. However, the claim of selective cellular Sirt3 inhibition by Compound **17**, as demonstrated by increased acetylation of the Sirt3 substrate MnSOD [195], is insufficiently supported because MnSOD acetylation was examined without comparison to total MnSOD protein levels [195]; therefore, the observed increase in MnSOD acetylation could be due to increased MnSOD protein levels unrelated to inhibition of Sirt3 deacetylase activity [195]. Therefore, further validation of the cellular activity of Compound **17** and similar mitochondrial-targeted Sirt3-selective probes will be necessary before employing them to dissect the roles of Sirt3 in health and aging-related diseases.

Consistent with the Sirt5 substrate preference for negatively-charged deacylation targets [229], derivatization of methyl thiocarbamoyl Sirt1-3 pseudopeptide inhibitors to carboxyethyl thiocarbamoyl resulted in a pseudopeptide inhibitor that inhibited Sirt5 with an IC_50_ value of 5 μM and exhibited 20- and 480-fold selectivity for Sirt5 over Sirt1 and Sirt6, respectively [184]. SAR studies based on thioglutaryl-lysine generated thiourea pseudopeptides [201]. The best of these pseudopeptides, succinyl thiocarbamoyl Compound **49** (Figure 5D), inhibited Sirt5 desuccinylation and deglutarylation with IC_50_ values of 170 nM and 110 nM, respectively [201]. Moreover, Compound **49** demonstrated >7-fold selectivity for Sirt5 over Sirt1/2/3/6 [201]. However, Compound **49** exhibited poor cellular activity [202]. As a result, the ethyl ester prodrug NRD167 (Figure 5D) was developed, which selectively decreased the proliferation of Sirt5-dependent acute myeloid leukemia (AML) cell lines with IC_50_ values of 5–8 μM, induced apoptosis at similar concentrations, and disrupted cellular oxidative phosphorylation and glycolysis, recapitulating the effects observed upon Sirt5 knockdown in Sirt5-dependent AML cells [202].

Despite being important for recognition by the Sirt5 substrate binding cleft [230], the carboxylate moiety of Compound **49** was hypothesized to be responsible for reduced cellular penetrance and activity (compare Compound **49** and NRD167 in Figure 5D) [202,229]. Succinyl thiocarbamoyl pseudopeptides containing carboxylate isosteres were developed to overcome this limitation [230]. The best compound, substituting the carboxylate for a masked tetrazole, penetrated cells and inhibited Sirt5-dependent AML cell line proliferation at half-maximal growth inhibitory concentrations (GI_50_) of 9–24 μM [230]. Additional SAR studies of Compound **49** focused on adding aryl fluorosulfate moieties to enhance inhibitor electrophilicity [231]. This class of thiourea pseudopeptides facilitated mechanism-based Sirt5 inhibition by forming covalent bonds at select Sirt5 tyrosine residues and producing inhibitor-Sirt5 covalent conjugates detectable via liquid chromatography–mass spectrometry [231]. Notably, aryl-fluorosulfate thiourea pseudopeptide Compound **17** covalently bound Sirt5 overexpressed in HEK293T cell lysates, native Sirt5 in living HeLa cells, and native Sirt5 in mouse cardiac tissue, demonstrating that covalent mechanism-based sirtuin inhibitors are an exciting new means to inhibit sirtuin activity [231].

Thiourea derivatives are an attractive class of isoform-selective sirtuin inhibitors with multiple practical advantages compared to thioacyl sirtuin inhibitors [184,190,225,228]. Moreover, these inhibitors harbor nanomolar potency, well-characterized cellular activity, and improved drug-like characteristics. Thus, thiourea derivatives exhibit high translational potential for investigating the therapeutic efficacy of selective sirtuin inhibition in a variety of cancers. Further preclinical studies in mouse models of cancer will aid further inhibitor optimization and begin to dissect the roles that specific sirtuins play in cancer pathogenesis.

### 4.5. Mechanism-Based Sirtuin Inhibition by Alternative Compounds

Some non-sulfur acetyl-lysine peptides and peptidomimetics also exhibit mechanism-based sirtuin inhibition [232,233,234]. For example, a fluorescent Sirt1 pseudopeptide substrate was modified to generate acetyl analogs containing electron-withdrawing or anion-stabilizing groups to increase inhibitor nucleophilicity and promote interaction with both the acylated substrate binding cleft and the NAD^+^ binding pocket [232]. The most potent acetyl analog identified was Compound **2k**, which contained an ethoxycarbonyl moiety at the α-carbon of the acetamide group [232]. Compound **2k** inhibited Sirt1 with an IC_50_ value of 3.9 μM and demonstrated 17- and >77-fold selectivity for Sirt1 over Sirt2 and Sirt3, respectively [232]. Mechanism-based inhibition was facilitated by the pseudopeptide analog forming a thermodynamically favored acetyl-lysine/ADP-ribose conjugate detectable via mass spectrometry [232]. However, the occupation of both the acyl-lysine and NAD^+^ binding pockets was not structurally confirmed [232]. Compound **2k** induced a concentration-dependent increase in acetylation of the Sirt1 substrate p53 in HCT116 colon cancer cells co-treated with etoposide, consistent with on-target cellular Sirt1 inhibition [232]. Carboxamides, isosteres of the acetyl group, have also demonstrated mechanism-based sirtuin inhibition [233,234]. In particular, when incorporated into peptides or pseudopeptides, the acetyl-lysine analog *L*-2-amino-7-carboxamidoheptanoic acid (L-ACAH) inhibited Sirt1-3 and yielded a long-lived catalytic intermediate detectable via mass spectrometry, suggesting mechanism-based sirtuin inhibition [233]. Derivatization of L-ACAH to ethyl L-ACAH increased inhibitory potency towards Sirt1-3 by ~2–7-fold, while carboxymethyl and dodecyl L-ACAH analogs were Sirt5 and Sirt6 inhibitors [234]. Although these non-sulfur-based sirtuin inhibitors generally exhibit weak potency and sirtuin isoform selectivity [232,233,234], they remain intriguing alternatives to thioamide-based compounds and warrant further investigation.

### 4.6. Mechanism-Based Sirtuin Inhibition with Cyclic Peptides

Another method to circumvent the limitations of peptide-based inhibitors [220] is cyclization to form cyclic peptides, which exhibit increased resistance to peptidases and improved cell permeability [235]. Cyclic peptides can also enhance target affinity and selectivity due to conformational restriction and maximization of favorable interactions with protein targets [235]. Therefore, cyclic peptides have increased translational potential compared to other sirtuin peptidic inhibitors. Linear thioacetyl-lysine Sirt1-3 peptide inhibitors were converted into monocyclic and bicyclic analogs, whereby several cyclized peptides exhibited increased Sirt1/2 inhibitory potency and/or isoform selectivity compared to the parent linear Sirt1-3 peptide inhibitors [191,192]. Monocyclic thioacetyl-lysine peptide Compound **10** (Table 3), one of the most potent Sirt2 inhibitors identified, inhibited Sirt2 with an IC_50_ value of 10.1 nM with ~seven- and eight-fold selectivity for Sirt2 over Sirt1 and Sirt3, respectively [191]. Consistent with the advantages of cyclic peptide inhibitors [235], a bicyclic Sirt1-3 thioacetyl-lysine peptide inhibitor exhibited increased proteolytic stability relative to the linear peptide inhibitor [192]. The bicyclic inhibitor also demonstrated on-target cellular activity, as demonstrated by increased acetylation of the Sirt1 substrate p53 in the HCT116 colon cancer cell line and decreased proliferation of a melanoma cell line that is susceptible to Sirt1/3 inhibition [192]. Additional optimization generated cyclic thioacetyl-lysine tripeptides which exhibited dual Sirt1/2 inhibition (IC_50_ = 220–890 nM) and ~4–8-fold selectivity for Sirt1/2 over Sirt3 [193]. Like previously developed cyclic peptide inhibitors [192], several of the dual Sirt1/2 inhibitors demonstrated increased proteolytic stability [193].

Cyclic peptide sirtuin inhibitors with thiourea warheads are also being explored [236,237,238]. For example, cyclic peptides with carboxyethylthiocarbamoyl warheads were designed to selectively target Sirt5 [236]; the best cyclic peptides inhibited Sirt5 with an IC_50_ value of ~2.2 μM and demonstrated ≥60-fold selectivity for Sirt5 over Sirt1/2/3/6 [236]. A cyclic peptide with a tetradecyl thiocarbamoyl warhead also inhibited Sirt6 with an IC_50_ value of 319 nM and ~20-, ~11-, and >940-fold selectivity for Sirt6 over Sirt2, Sirt3, and Sirt5, respectively; however, the inhibitor exhibited only ~two-fold selectivity for Sirt6 over Sirt1 [238]. Cyclic peptides containing thiourea and carboxamide warheads were also developed as the first mechanism-based Sirt7 inhibitors [237]. These cyclic peptides inhibited tRNA-activated Sirt7 deacetylase activity (IC_50_ = 5–11.1 μM), yet inhibited Sirt1/2/3/6 with similar or increased potency (IC_50_ = 0.55–11.2 μM) [237]. Nonetheless, these cyclic peptides provide a strong foundation for future optimization to increase sirtuin isoform selectivity as additional structural and biochemical data for Sirt7 become available.

## 5. Peptidic Non-Mechanism-Based Sirtuin Inhibitors

**Figure 6:**
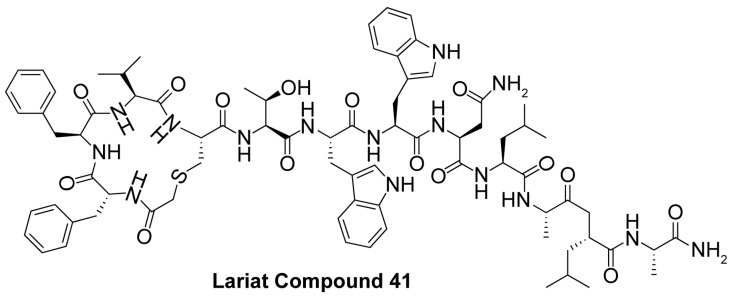
Peptide lariats represent a novel class of peptidic non-mechanism-based sirtuin inhibitors. Peptide lariat Compound **41** [241].

The first cyclic peptide sirtuin inhibitors, inspired by prior studies with linear peptides in yeast, slowed sirtuin deacylation without chemically modifying the sirtuin active site [239]. Previously, trifluoroacetyl-lysine warheads were found to bind sirtuins with ~six-fold greater affinity than acetyl-lysine and slow the rate of nicotinamide formation by ~five orders of magnitude compared to thioacetyl-lysine warheads and acetyl-lysine [25,176,240]. This inspired the use of a cell-free translation and peptide screening platform to generate peptides containing trifluoroacetyl-lysine warheads as Sirt2 inhibitors [239]. This screen generated two cyclic peptide inhibitors with trifluoroacetyl-lysine warheads that inhibited Sirt2 with IC_50_ values of 3.2–3.7 nM and demonstrated ~9–150-fold selectivity for Sirt2 over Sirt1/3 [239]. Recently, the same cell-free translation peptide screen used to identify the first cyclic peptide inhibitors [239] was employed to develop non-mechanism-based cyclic peptide and peptide lariat sirtuin inhibitors [241]. Peptide lariat Compound **41** (Figure 6) was found to be a novel Sirt7 inhibitor (IC_50_ = 2.7 μM) that exhibited ~55-fold selectivity for Sirt7 over Sirt6, with minimal impacts on Sirt1/2/3/5 at 10 μM [241]. HEK293T cells treated with Compound **41** demonstrated increased acetylation of the Sirt7 substrate histone H3K18, consistent with on-target cellular Sirt7 inhibition [241]. Compound **41** represents a substantial improvement in Sirt7 inhibitor potency and sirtuin isoform specificity [237,241]. Therefore, Compound **41** demonstrates that peptide lariats are an exciting new avenue for sirtuin inhibitors, especially since their precise mode of sirtuin inhibition is unknown [241].

## 6. Conclusions

To aid investigators studying the cellular functions of sirtuin isoforms, we recommend several compounds (Table 4) for use as chemical probes and foundations for development into more effective therapeutics, while considering their limitations. Notably, no activators of Sirt2, Sirt4, or Sirt7 have been reported (Table 4). The suggested Sirt1 activators SRT1720 or SRT2104 [59,60] show high potency, isoform selectivity, and biological activity in preclinical and early clinical trials. However, SRT1720 and SRT2104 efficacy is limited to scenarios where Sirt1 substrates include hydrophobic and aromatic amino acids’ *C*-terminal to the acyl-lysine residue [39,86,87]. Moreover, SRT1720 and SRT2104 exhibit off-target effects on non-sirtuin proteins, including AMPK, receptors, enzymes, transporters, and ion channels [85,90]. In contrast, due to its unique inhibition mechanism, the Sirt1 inhibitor EX-527 [127] has nanomolar potency and translational potential in Huntington’s disease but is often used in cellular studies at micromolar concentrations that also inhibit Sirt2 [147,148,149,150]. The recommended Sirt2 inhibitors TM [186], AF8/AF10 [190], and SirReal2 [124] display nanomolar potency, high isoform selectivity, and efficacy in various cancer models. However, further optimization of compound solubility is required for TM and AF8/AF10 [186,190].

The suggested Sirt3 activator ADTL-SA1215 [62] possesses high potency and is effective in cell and mouse models of breast cancer. However, this Sirt3 activator was only recently reported [62], and follow-up studies from other laboratories using this activator have yet to be published. On the other hand, mitochondrially targeted myristoyl-thiocarbamoyl pseudopeptides like YC8-02 [194] and Compound **17** [195], the recommended Sirt3 inhibitors, demonstrate a first-in-class ability to target a sirtuin isoform within a subcellular organelle. Nonetheless, these inhibitors lack selectivity for Sirt3 over Sirt1/2 in vitro, so further confirmation of mitochondrial-targeting and selective Sirt3 inhibition is required at the cellular level for both compounds [194,195]. The suggested Sirt4 inhibitors Compounds **60** and **69** [132] are first-in-class modulators of Sirt4 deacylase activity [132]. Sirt4 inhibitor development would be accelerated by in-depth structural and kinetic studies confirming the modes by which Compounds **60** and **69** bind and inhibit Sirt4, which have not been reported [132].

The suggested Sirt5 activator, 1,4-dihydropyridine derivative Compound **30**, exhibits good selectivity for Sirt5 [63]; however, like ADTL-SA1215, the efficacy of this compound in the hands of others remains to be tested [63]. Succinyl-thiocarbamoyl peptides like Compound **49** [201] and NRD167 [202], the recommended Sirt5 inhibitors, exhibit nanomolar potency and efficacy in Sirt5-dependent acute myeloid leukemia cell lines. However, the effects of these inhibitors in mouse or other preclinical models of acute myeloid leukemia have not yet been reported [201,202]. The suggested Sirt6 activators LPA [65] and MDL-811 [107] substantially increase Sirt6 deacetylase activity. In particular, MDL-811 exhibits high efficacy in mouse models of cancer and neural damage [107]. In contrast to typical sirtuin inhibition modes, the recommended Sirt6 inhibitor, JYQ-42 [165], allosterically decreases Sirt6 deacetylase activity and demonstrates translational promise in pancreatic cancer cell models. However, SAR optimization of JQY-42 has yet to be conducted [165], suggesting that enhancing JQY-42 binding to the unique Sirt6 pocket Z will be necessary to increase its inhibitory potency from the micromolar to the nanomolar range. Despite requiring further optimization to increase overall potency and selectivity [239], the recent development of peptide lariat Compound **41**, the recommended Sirt7 inhibitor [239], opens up an exciting avenue of peptide lariat sirtuin inhibitor research.

With universal cellular expression yet unique subcellular localizations and functions [10,11], sirtuins are paradoxically reported to promote and prevent aging-related diseases [46,47,48]. Existing small molecule activators, small molecule inhibitors, and mechanism-based inhibitors of sirtuins allow unprecedented insight into the kinetics of sirtuin deacylase activity and provide rationales for the selective activation or inhibition of sirtuins in particular biological and disease contexts. As the sirtuin field evolves, continued efforts to enhance potency, isoform specificity, cellular activity, solubility, and pharmacokinetic properties will allow for further dissection of individual sirtuin isoform roles in the pathophysiology of aging. Moreover, such studies will lead to the identification of improved scaffolds for developing safe, bioactive, sirtuin-selective drugs with therapeutic benefits for aging-related diseases.

## Figures and Tables

**Table 1:** Select activators of sirtuin deacylase activity.

Compound Name	Structure	Targeted Sirtuin Isoform	Potency (EC_50_), μM	Fold Activation	Cell Active	Citations
Resveratrol	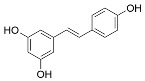	Sirt1	~100	13.4	Yes	[58,59]
SRT1720	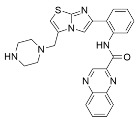	Sirt1	~0.10	~8	Yes	[59]
SRT2104	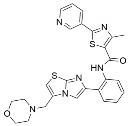	Sirt1	0.43 ^a^	~2	Yes	[60]
Honokiol	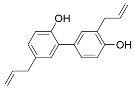	Sirt3	N.D.	~2	Yes	[61]
ADTL-SA1215	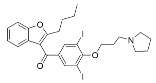	Sirt3	0.21	2	Yes	[62]
Compound **31**	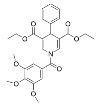	Sirt3	~100–200	~5–1000	Yes	[63]
Compound **30**	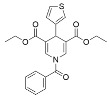	Sirt5	~40	~3–5	Yes	[63]
UBCS039	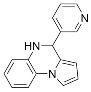	Sirt6	38	2	Yes	[64]
LPA	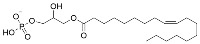	Sirt6	25	48	Yes	[65]
MDL-811	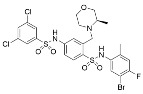	Sirt6	7.1	44	Yes	[66]
Compound **12q**	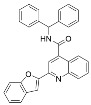	Sirt6	8.9 * 5.4 **	~12–18 * ~40 **	Yes	[67]

^a^ = potency as assessed using EC_1.5_. N.D. = potency not determined. * = demyristoylase activity. ** = deacetylase activity.

**Table 2:** Select small molecule inhibitors of sirtuin deacylases.

Compound Name	Structure	Targeted Sirtuin Isoform	Potency (IC_50_), μM	Selectivity	Cell Active	Citations
ELT-31	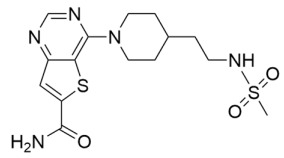	Sirt1/2/3	0.001–0.033	Nonselective	N.D.	[126]
EX-527	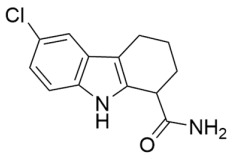	Sirt1	0.12	~20–800 fold over Sirt2/3	Yes	[127]
Compound **86**	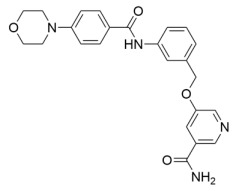	Sirt2	0.02	~100–400 fold over Sirt1/3	Yes	[128]
AGK2	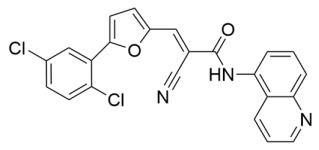	Sirt2	3.5	>14 fold over Sirt1/3	Yes	[129]
AK7	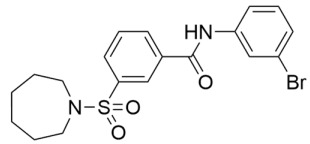	Sirt2	15.5	>4 fold over Sirt1/3	Yes	[130]
SirReal2	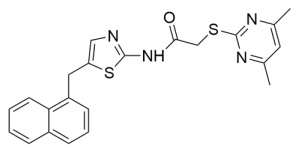	Sirt2	0.14	>1000 fold over Sirt1/3/4/5/6	Yes	[124]
MIND4	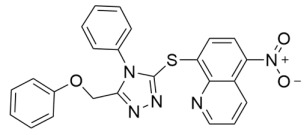	Sirt2	3.5	Selective over Sirt1/3	Yes	[131]
Compound **60**	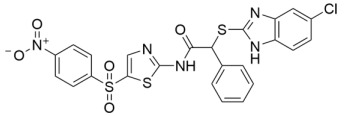	Sirt4	0.9	Non-selective over Sirt2; ~3.5–5.5 fold over Sirt1/3/5/6	Yes	[132]
Compound **69**	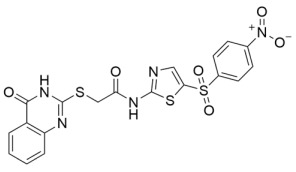	Sirt4	16	~2–3 fold over Sirt1/2/3/5/6	Yes	[132]
Compound **47**	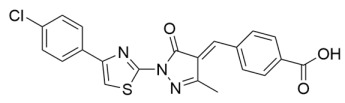	Sirt5	0.21	>3800 fold Sirt5 over Sirt1/2/3/6	N.D.	[133]

N.D. = cellular activity not determined.

**Table 3:** Select mechanism-based inhibitors of sirtuin deacylase activity.

Lysine Derivative	Warhead	Targeted Sirtuin Isoform(s)	Potency (IC_50_), μM	Selectivity	Cell Active	Citations
Thioacetyl	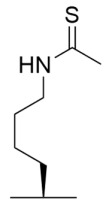	Sirt1/2/3	0.18–29.4	Largely non-selective	Yes	[175,176,177,178,179,180,181,182,183]
Thiocarbamoyl	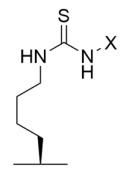	Sirt1/2/3	1.7–159.1	Non-selective	Yes	[184,185]
Thiomyristoyl (TM)	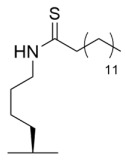	Sirt2	0.03–0.09 * 0.04–3.3 **	Up to ~3500–7100 fold over Sirt1/3/5/6/7	Yes	[186,187,188]
Soluble TM	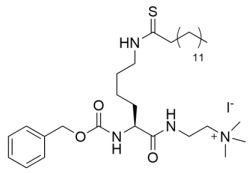	Sirt2	0.03	~72–94 fold over Sirt1/3; >1500 fold over Sirt6	Yes	[189]
Fatty acyl thiocarbamoyl	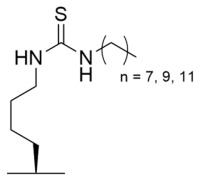	Sirt2	0.06–0.15	~180–2500 fold over Sirt1/3	Yes	[190]
Cyclic thioacetyl	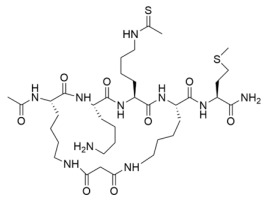	Sirt1 Sirt2	0.002 0.01	19–62 fold over Sirt2/3; 7–8 fold over Sirt1/3	Yes	[191,192,193]
Mitochondrially-targeted TM	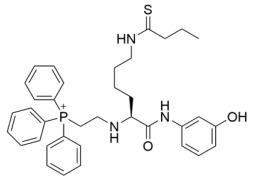	Sirt3	0.53	N.D.C.	Yes	[194]
Mitochondrially-targeted thiocarbamoyl	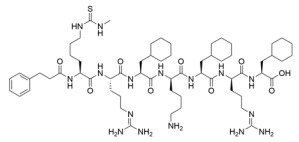	Sirt3	1.1	N.D.C.	Yes	[195]

N.D.C. = isoform selectivity not determined in cells * = deacetylase activity ** = demyristoylase activity.

**Table 4:** Recommended sirtuin modulators for cellular studies.

Targeted Sirtuin Isoform	Activators	Inhibitors
Sirt1	SRT1720 [59], SRT2104 [60]	EX-527 [127]
Sirt2	N/A *	TM [186], AF8/10 [189,190], SirReal2 [124]
Sirt3	ADTL-SA1215 [62]	Mitochondrially targeted myristoyl-thiocarbamoyl pseudopeptides [194,195]
Sirt4	N/A *	Compound **60**/**69** [132]
Sirt5	1,4-dihydropyridine derivative Compound **31** [63]	Succinyl-thiocarbamoyl pseudopeptides [201,202]
Sirt6	LPA [65], MDL-811 [107]	N/A *
Sirt7	N/A *	Peptide lariat Compound **41** [240]

N/A * indicates not available, where no suitable sirtuin modulator is recommended.

## Data Availability

No new data were created or analyzed in this study. Data sharing is not applicable to this article.

## Abbreviations

AceCS2 (acetyl-CoA synthetase 2); AML (acute myeloid leukemia); AMPK (AMP-activated protein kinase); BET (bromodomain and extraterminal domain); BIM (bisindolylmaleimide); de-HMGylation (de-β-hydroxy β-methylglutarylation); DLBCL (diffuse large B cell lymphoma); EC50 (half-maximal effective concentration); eIF5a (eukaryotic translation initiation factor 5a); FFA (free fatty acid); FOXO3a (fork-head box O3a); GI50 (half-maximal growth inhibitory concentration); GOT1 (glutamate oxaloacetate transaminase 1); GSK (GlaxoSmithKline); HCC (hepatocellular carcinoma); HDAC (histone deacetylase); HNSCC (head and neck squamous cell carcinoma); IC50 (half-maximal inhibitory concentration); IDO1 (indoleamine 2,3-dioxygenase 1); Ki (inhibition constant); KM (Michaelis constant); L-ACAH (L-2-amino-7-carboxamidoheptanoic acid); LFA (long-chain fatty acid); LPA (oleoyl-lysophosphatidic acid); MetAP2 (methionine aminopeptidase 2); MnSOD (manganese superoxide dismutase); NAD (nicotinamide adenine dinucleotide); NRF2 (nuclear factor erythroid 2-related factor 2); NSCLC (non-small cell lung cancer); OAADPr (O-acyl-ADP-ribose); OSCC (oral squamous cell carcinoma); OSCP (oligomycin-sensitivity conferring protein); PARP (poly-ADP-ribose polymerase); PDHA1 (pyruvate dehydrogenase subunit A1); PGC-1α (proliferator-activated receptor γ coactivator 1α); SAR (structure–activity relationship); SBD (STAC-binding domain); SirReal (sirtuin-rearranging ligand); Sir2 (Silent Information Regulator 2); STAC (sirtuin-activating compound); TM (thiomyristoyl); TPP+ (triphenylphosphonium); TRPM2 (transient receptor potential cation channel, subfamily M, member 2); TS (thiosuccinyl); maximal reaction velocity (Vmax).
